# Strategies in regulating glioblastoma signaling pathways and anti-invasion therapy

**DOI:** 10.1371/journal.pone.0215547

**Published:** 2019-04-22

**Authors:** Eunok Jung, Aurelio A. de los Reyes V, Kurt Jan A. Pumares, Yangjin Kim

**Affiliations:** 1 Department of Mathematics, Konkuk University, Seoul, Republic of Korea; 2 Institute of Mathematics, University of the Philippines Diliman, Quezon City, Philippines; 3 Mathematical Biosciences Institute and Department of Mathematics, Ohio State University, Columbus, Ohio, United States of America; University of Pécs Medical School, HUNGARY

## Abstract

Glioblastoma multiforme is one of the most invasive type of glial tumors, which rapidly grows and commonly spreads into nearby brain tissue. It is a devastating brain cancer that often results in death within approximately 12 to 15 months after diagnosis. In this work, *optimal control theory* was applied to regulate intracellular signaling pathways of miR-451–AMPK–mTOR–cell cycle dynamics via glucose and drug intravenous administration infusions. Glucose level is controlled to activate miR-451 in the up-stream pathway of the model. A potential drug blocking the inhibitory pathway of mTOR by AMPK complex is incorporated to explore regulation of the down-stream pathway to the cell cycle. Both miR-451 and mTOR levels are up-regulated inducing cell proliferation and reducing invasion in the neighboring tissues. Concomitant and alternating glucose and drug infusions are explored under various circumstances to predict best clinical outcomes with least administration costs.

## Introduction

Glioblastoma multiforme (GBM) is the most common and the most aggressive type of brain cancer. The median length of survival time is approximately 12 to 15 months following diagnosis. GBM is characterized by anaplasia, nuclear atypia, cellular pleomorphism, mitotic activity, and more importantly, alternating phases of rapid proliferation and aggressive invasion into the surrounding brain tissue. This leads to an inevitably critical recurrence even after the surgical resection of the main tumor mass [1, 2]. The mainstay of treatment for GBM is surgery, followed by radiotherapy and chemotherapy. Despite advances in these approaches, glioma cells can still invade the neighboring tissues beyond detection leading to tumor recurrence. High probability of main treatment failure also encourages researchers to investigate the use of innovative treatments when the first line of therapy has failed, in order to improve clinical outcomes [3].

In the tumor microenvironment (TME), glioma cells encounter many challenges including hypoxia, acidity, and limited nutrient availability. To maintain rapid growth, tumor cells need to adapt to these biochemical changes and modify their metabolic activity by increasing glycolysis even in the presence of oxygen. This process is called the *Warburg effect* which requires consuming considerable amounts of glucose [4]. The tricarboxylic acid cycle, or *Krebs cycle*, plays an important role in the breakdown of organic fuel molecules and the survival in non-hypoxic normal differentiated cells. These molecules include glucose, fatty acids, and some amino acids. While differentiated cells favor this type of metabolism, which is very efficient in terms of ATP production, tumor cells adopt the inefficient aerobic glycolysis producing relatively large amounts of waste product in the form of lactic acid [5]. This may provide cancer cells the advantage of not having to depend on oxygen as an energy source especially in a hostile tumor microenvironment, thus leading to longer survival [6]. Inhibition of glycolysis may also prevent drug resistance thus a better understanding of this metabolic pathway may lead to better treatment options and clinical outcomes [7]. Developing strategies of metabolic adaptation, angiogenesis, and migration is critical for cancer cells in order to survive metabolic stress and ensure enough nutrient supply as tumor mass accumulates where glucose supply may fluctuate due to heterogeneous biochemical and biophysical conditions [8]. Therefore, adequate cellular responses to glucose withdrawal are critical for cancer cell survival. Cancer cells then activate the 5′-adenosine monophosphate activated protein kinase (AMPK) pathway under metabolic stress. It is the master cellular sensor of energy availability which enhances glucose uptake and conserve energy, thus avoiding cell death [9]. MicroRNAs, also abbreviated as miRNA, are approximately 22 nucleotide single-stranded non-coding ribonucleic acids (RNAs) that are known to regulate gene expression [10]. Dysregulation of microRNA expression has been linked to oncogenic and tumor suppressor activities in several types of cancer, including GBM [11, 12], where altered miRNA expression contributes to tumorigenesis [13, 14].

Godlewski *et al*. [8] identified an interesting mechanism of glioma cell migration and proliferation wherein a particular microRNA, miR-451, and its counterpart, AMPK complex (CAB39/ LKB1/STRAD/AMPK), determine whether the cell favors growth at the expense of invasion or conversely. Moreover, they also identified a potential feedback loop between LKB1 and miR-451 allowing for a sustained and robust response to glucose withdrawal [15]. It was found out that (i) under high (normal) glucose conditions, up-regulation of miR-451 leads to the down-regulation of AMPK complex, which then leads to elevated proliferation and decreased migration of glioma cells and (ii) glucose withdrawal induces down-regulation of miR-451 and up-regulation of AMPK, which promotes cell migration with reduced proliferation.

The mathematical models developed by Kim *et al*. [14, 16, 17] describe the effects of the miR-451–AMPK core control system on cell proliferation and migration in glioblastoma. It explains the response of miR-451 to high and low glucose levels as well as the mutual antagonism between miR-451 and AMPK complex concentrations. Kim *et al*. [18] then extended the model to include the dynamics of mammalian target of rapamycin (mTOR), which is a protein kinase that links with other proteins as well as to the cell cycle dynamics, to form the miR-451–AMPK–mTOR core control system. This mutual antagonism between miR-451 and AMPK, which was predicted in these mathematical models [14, 16–18], was confirmed by recent experiments. For example, Ansari *et al*. [19] recently found that (i) the miR-451 transcription in GBM cells is induced by unrestricted activity of its transcription factor OCT1 (official gene symbol POU2F1) in the presence of abundant glucose, resulting in AMPK inhibition through direct targeting of CAB39 in the LKB1 complex; and (ii) the miR-451 level is inhibited through the phosphorylation and inactivation of OCT1 at S335 by AMPK in response to glucose depletion-induced metabolic stress, leading to a reciprocal negative feedback loop between miR-451 and AMPK. In this case, suppression of miR-451 in turn leads to sustained AMPK activities and a robust response to glucose withdrawal in GBM cells. The multiscale mathematical models [14, 16–18] also predict the growth–invasion cycling patterns of glioma cells in response to fluctuating glucose uptake in the tumor microenvironment. The core control system predicts bistability and hysteresis bifurcation when delayed down-regulation of miR-451 activities along certain molecular pathways would induce glioma cells to stay longer in the proliferative phase despite relatively low glucose concentrations, making this mechanism a therapeutic target.

The cell cycle represents an integrated series of events that regulates complex processes including cell proliferation, cell division and DNA replication, regulated by a complex hierarchy of genetic and metabolic networks which involves several transition states of varied lengths and checkpoints [20]. The stages of the cell cycle are as follows: (i) synthesis phase (S), a period where DNA replication occurs; (ii) gap phase 2 (G2), during which proteins required for mitosis are produced; (iii) mitosis phase (M), a period where chromatin condensation, nuclear envelope breakdown (NEBD), chromatid separation, and cytokinesis happens; (iv) gap phase 1 (G1), in which genes necessary for DNA replication are activated and the protein agents of S phase progression are accumulated; and (v) resting phase (G0), a state in which cells can exit the cell cycle and enter a phase of quiescence or relative inactivity [21]. The progression of mammalian cell cycle is tightly regulated by coordinated activation of cyclin-dependent kinases (CDKs) family [22]. The CDKs are positively regulated by cyclins and negatively by CDK inhibitors (CDKIs) such as the proteins p15, p16, p21 and p27. In cancer cells, cyclins are over-expressed while CDKIs are under-expressed which results in the dysregulation of the cell cycle, and promoting uncontrolled cell growth [20]. Tyson and Novak [23, 24] identified that the transition between two stable steady states, G1 and S–G2–M cell-cycle phases, are described using the kinetic relations of the model that is controlled by changes in cell mass.

A standard GBM treatment is surgery followed by chemotherapy and radiotherapy. However, even under best circumstances, the mean survival of this disease is about a year. Poor outcomes of standard care treatments are due to the topographically diffuse nature of the disease [25]. By the time of diagnosis, typical GBM cells may have widely spread throughout the brain tissue [26–28], increasing the potential of recurrence. Thus, annihilation of distant tumor satellites is implausible despite surgically removing all the essential tumor seen on enhanced MRI scan [29]. Knowing the exact margins of a tumor mass in real patients is indeed a daunting task. In this study, it is assumed that a major tumour mass has been surgically removed and that the infiltrative tumor cells are near the surgical site. The objective is to prevent the glioma cells from further diffusing into the surrounding brain tissue. Localization approach will be utilized, that is, glioma cell invasion will be blocked keeping them in a proliferative phase while also attempting to limit excessive growth before a second surgery [17]. Our analytical tool is primarily based on the framework of *optimal control theory*, which has been successfully used to make informed decisions involving biological models such as optimal treatment strategies in human immunodeficiency virus (HIV) models [30–33], tuberculosis [34–36], and cardiopulmonary resuscitation (CPR) techniques [37, 38]. Optimal control theory is also applied to the miR-451–AMPK core control system to determine the intravenous glucose and/or drug infusion protocols with least possible cost under various circumstances in de los Reyes, *et al*. [39].

Recently, Kim *et al*. [40] developed an intracellular signaling pathway model that extends the miR-451–AMPK–mTOR core control system including the cell cycle dynamics. In this work, a potential control problem is formulated in order to maintain high levels of miR-451 and mTOR (low levels of AMPK) inducing cell proliferation prohibiting cell motility and invasion to the neighboring tissues. With glucose levels as a key regulator of miR-451 activity which also activates mTOR in the downstream signaling pathway, glucose intravenous infusion is considered to up-regulate miR-451 and mTOR concentrations above certain threshold values. In addition, a drug suppressing the inhibitory effect of mTOR by the AMPK complex is incorporated. This drug can be administered concomitantly or alternately with glucose as a secondary intravenous infusion. The controls are then given by dose rates of glucose and drug intravenous administrations regulating upstream and downstream signaling pathway, respectively. Solution of the optimal control problem aims to determine infusion protocols with minimal glucose and drug amount, and least administration costs. Hence, glucose and drug levels are regulated to prevent rapid tumour growth, hyperglycemia, and further drug complications. The results propose plausible glucose and drug intravenous infusion controls which indicate the time of administration, frequency, number of administrations, and dosages of glucose and drug per infusion.

In the current study, we will first present the miR-451–AMPK-mTOR–cell cycle intracellular signalling pathway developed by Kim *et al*. [40]. A drug module is incorporated to up-regulate mTOR activities inducing cell proliferation. Then an optimal control problem is formulated with the goal of activating miR-451 and mTOR levels through glucose and drug intravenous infusions. Two different infusion protocols will be explored, namely, concomitant and alternating glucose and drug intravenous administrations. Optimal solutions for two strategies are presented and results on frequency, dosage per infusion, total glucose and drug amount, and relative cost incurred in the administrations are compared. The conclusion section discusses and summarizes the optimal control results, and provides outlook for future research directions.

## Materials and methods

### Intracellular signaling dynamics model

**Fig 1:**
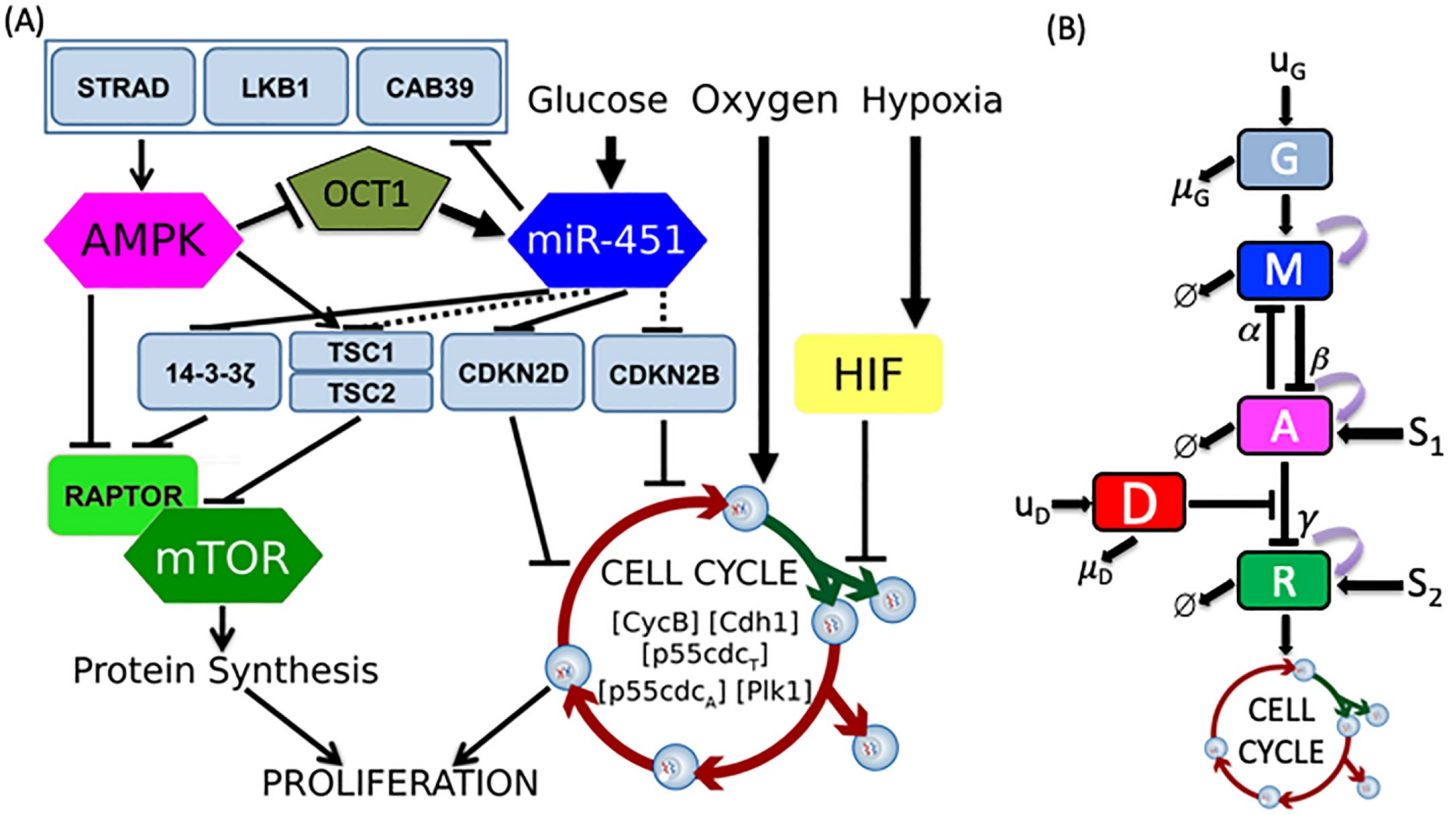
A model of the miR-451–AMPK–mTOR–cell cycle signaling pathway. (A) Detailed schematic diagram of cellular decision of proliferation and migration in glioblastoma [40]. (B) Block diagram of the theoretical model representing glucose (*G*) regulation on miR-451 (*M*), AMPK (*A*), mTOR (*R*) with the signaling pathway to the cell cycle dynamics and the drug (*D*) suppressing the inhibition of mTOR by AMPK.

In this section, we present the basic components of intracellular signalling pathway of tumor cell containing the core control miR-451–AMPK–mTOR and cell cycle pathway developed in Kim *et al*. [40], incorporating a drug which blocks the inhibitory pathway of mTOR by AMPK complex. The core control model identifies a key mechanism which determines the molecular switches between the proliferative and migratory phases in response to fluctuating glucose and drug levels. The simplified signaling pathways consists of five key determinants of the intracellular structure, namely, glucose level *G*, miR-451 level *M*, AMPK complex activity *A*, mTOR concentration *R*, and drug level *D*. The intracellular cell cycle dynamics are developed by Tyson and Novak [23, 24] including only the essential interactions for its regulation and control. The model captures the kinetics of chemical processes within the cell such as production, destruction, and different molecule interactions. The transition between two main steady states, G1 and S–G2–M phases, of the cell cycle are described using the kinetic relations of the model that is controlled by changes in cell mass. Powathil *et al*. [41] modified the model by using equivalent mammalian proteins, namely the Cdk-cyclin B complex [*CycB*], the APC-Cdh1 complex [*Cdh*1], the active form of the p55cdc-APC complex [*p*55*cdc*_*A*_], the total p55cdc-APC complex [*p*55*cdc*_*T*_], the active form of Plk1 protein [*Plk*1] and the cell mass [*mass*]. Also included are the effects of the changes in oxygen dynamics at the macroscopic level through the activation and inactivation of HIF-1 *α*. This results in changes in cell cycle length. In addition, the cell cycle inhibitory effect of p21 or p27 genes of HIF-1 *α* is incorporated. Kim *et al*. [40] proposed to link both the miR-451–AMPK–mTOR control system and the cell cycle dynamics to provide a mechanism driving the cell cycle to undergo the *quiescent* stage G0-phase depending on the concentration level of mTOR. Fig 1A depicts the detailed schematic diagram of the intracellular signalling networks including miR-451, AMPK complex, mTOR, and key players in the cell cycle module (CycB, Cdh1, p55cdcT, p55cdcA, Plk1). Kinetic interpretation of arrows and hammerheads represent induction and inhibition in the signaling network, respectively. The dimensionless diagram of the core control miR-451, AMPK complex, and mTOR linking to the cell cycle is depicted in Fig 1B. It should be noted that *u*_*G*_ and *u*_*D*_ are the sources of glucose and drug with decay rates *μ*_*G*_ and *μ*_*D*_, respectively, which can be controlled exogenously. *S*_1_ and *S*_2_ are signaling sources to AMPK complex and mTOR, respectively, while *α*, *β* and *γ* are inhibition strengths, and *ϕ* denotes decay.

**Fig 2:**
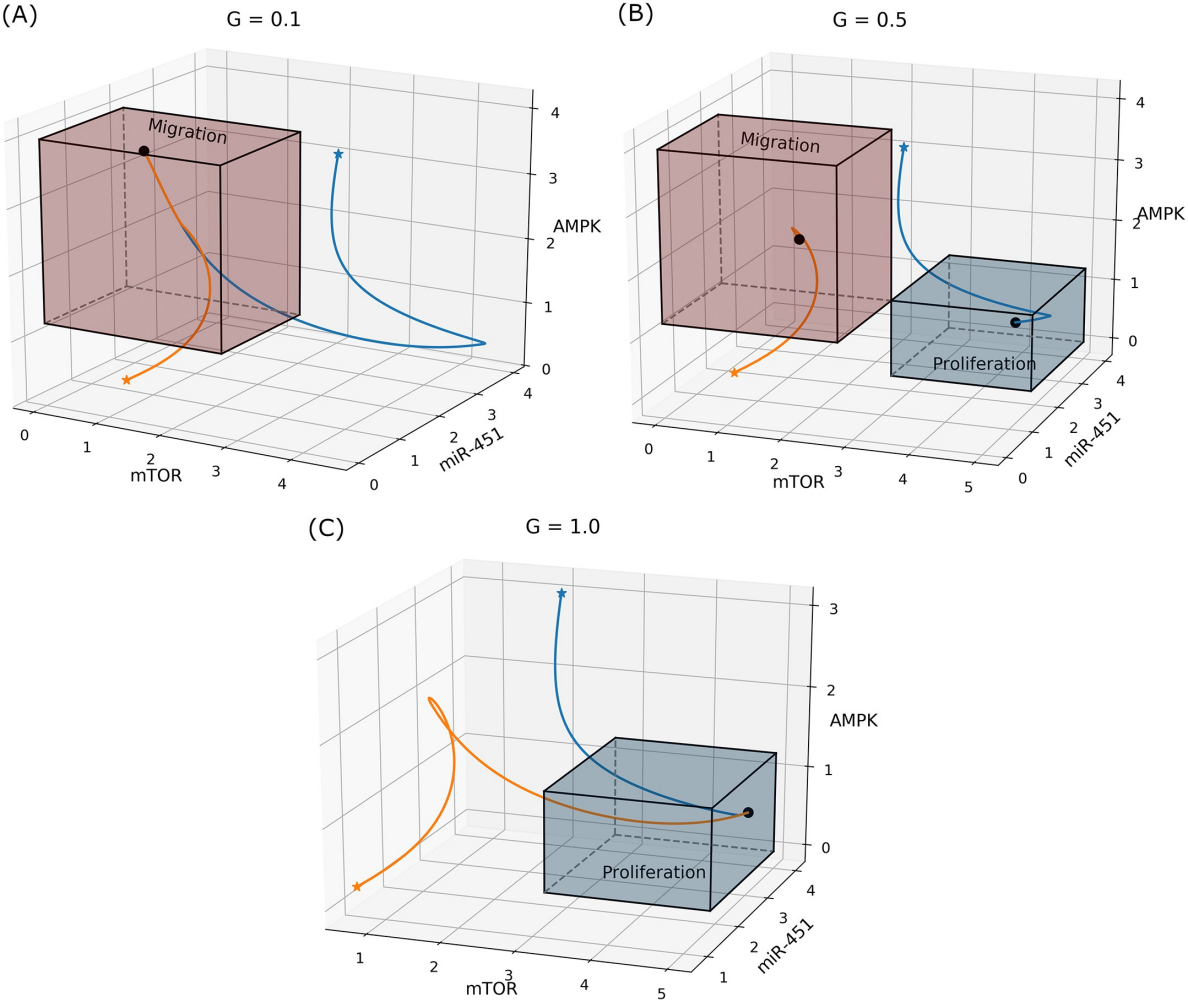
Effect of glucose on regulation of the core control system. Two trajectories of core control control concentrations in miR-451–AMPK–mTOR space in response to (A) low (*G* = 0.1), (B) intermediate (*G* = 0.5), and (C) high (*G* = 1.0) glucose levels.

It has been shown that high (normal) glucose concentration yields over expression of miR-451 levels (down-regulation of AMPK complex and up-regulation of mTOR) leading to elevated cell proliferation and reduced migration, while low glucose levels leads to down-regulation of miR-451 activities (up-regulation of AMPK complex and down-regulation of mTOR) reducing cell proliferation and inducing migration into the surrounding brain tissue [8, 15, 18, 40]. The effect of various glucose levels in the regulation of the core control is depicted in Fig 2.

Kim *et al*. [14] developed a core control system of glioma cell migration and proliferation by using a regulatory network of key molecules (miR-451 (*M*), AMPK (*A*)) as follows:

$$\begin{array}{c} \begin{array}{cc} \frac{dM}{dt} & =G+\frac{\ell_{1}\ell_{2}^{2}}{\ell_{2}^{2}+\alpha A^{2}}-M,\\ \frac{dA}{dt} & =\frac{1}{\epsilon_{1}}(S_{1}+\frac{\ell_{3}\ell_{4}^{2}}{\ell_{4}^{2}+\beta M^{2}}-A). \end{array} \end{array}$$ (1)

As observed in experiments [8, 15, 19], the regulatory system in Eq (1) includes a mutually antagonistic loop between miR-451 and AMPK complex in response to high and low glucose levels (*G*). This genetic toggle switch induces a monostable and bistable system, characterizing proliferation and critical cell infiltration of GBM cells in brain [14]. In the follow-up studies [14, 16–18] including mTOR (*R*) and cell cycle modules, this mutual antagonism and bistable system played a critical role in developing anti-invasion strategies. A mutually antagonistic feedback loop has been well-studied for its bistable properties by use of mathematical models ([42–45] and other references in [45]). For instance, in a study on a genetic toggle switch in Escherichia coli [42], a mutually inhibitory loop between repressor 1 and repressor 2 was shown to induce bistability in addition to monostable status. In particular, Lu *et al*. [45] in a theoretical study on miR-based regulation showed that the regulatory network including a mutual inhibition feedback circuit between the miR-34/SNAIL and the miR-200/ZEB can induce a tristable circuit of epithelial-hybrid-mesenchymal fate differentiation. A detailed analysis on the self-activating, tristable state-inducing toggle switch or more general multistable genetic circuits can be found in [43, 44]. Other biological networks without mutual inhibition can also induce a bistable system. For instance, Aguda *et al*. [11] investigated the role of miRNAs in regulation of cell cycle and *cancer zone* by emerging bistable toggle switch in the feedback loops of miR-17-92, E2F, and Myc.

**Fig 3:**
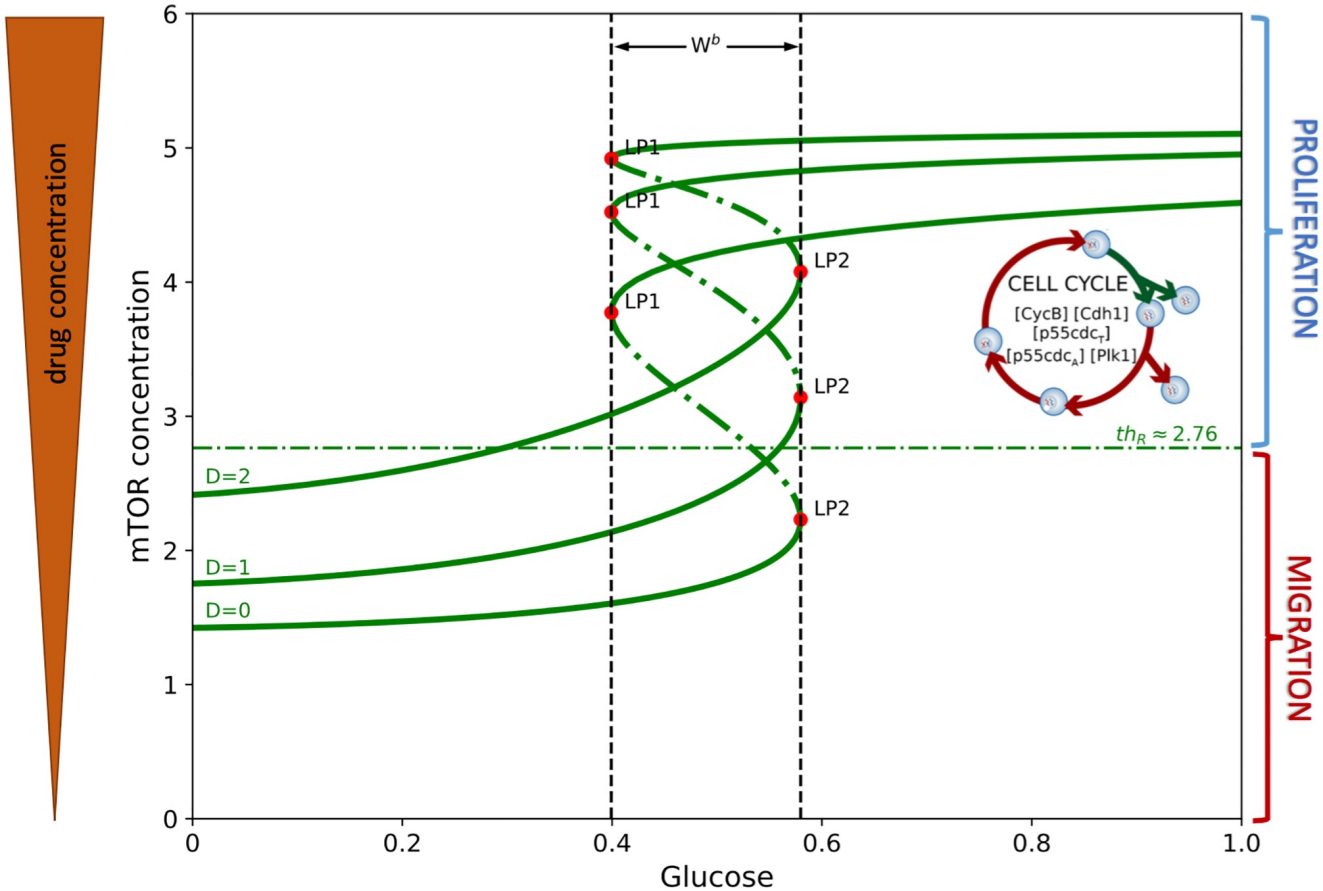
Bifurcation diagram of mTOR. Hysteresis diagram of mTOR concentration over the range of glucose levels and the corresponding effect of different drug concentrations on its dynamics.

In this study, we consider a drug *D* suppressing the inhibitory effect of mTOR by AMPK complex where the inhibition strength is given by *ζ*(*D*) = *e*^−*D*^. When *D* is large, *γe*^−*D*^ is small which makes *dR*/*dt* to be large. Thus, the presence of drug up-regulates mTOR activity that could eventually lead to cell proliferation. Fig 3 illustrates the mTOR bifurcation curve. Observe that increasing drug concentration shifts the hysteresis curve upwards keeping the same bistability window. Hence, with higher drug levels for the same glucose concentrations, mTOR is activated prompting elevated cell growth.

**Fig 4:**
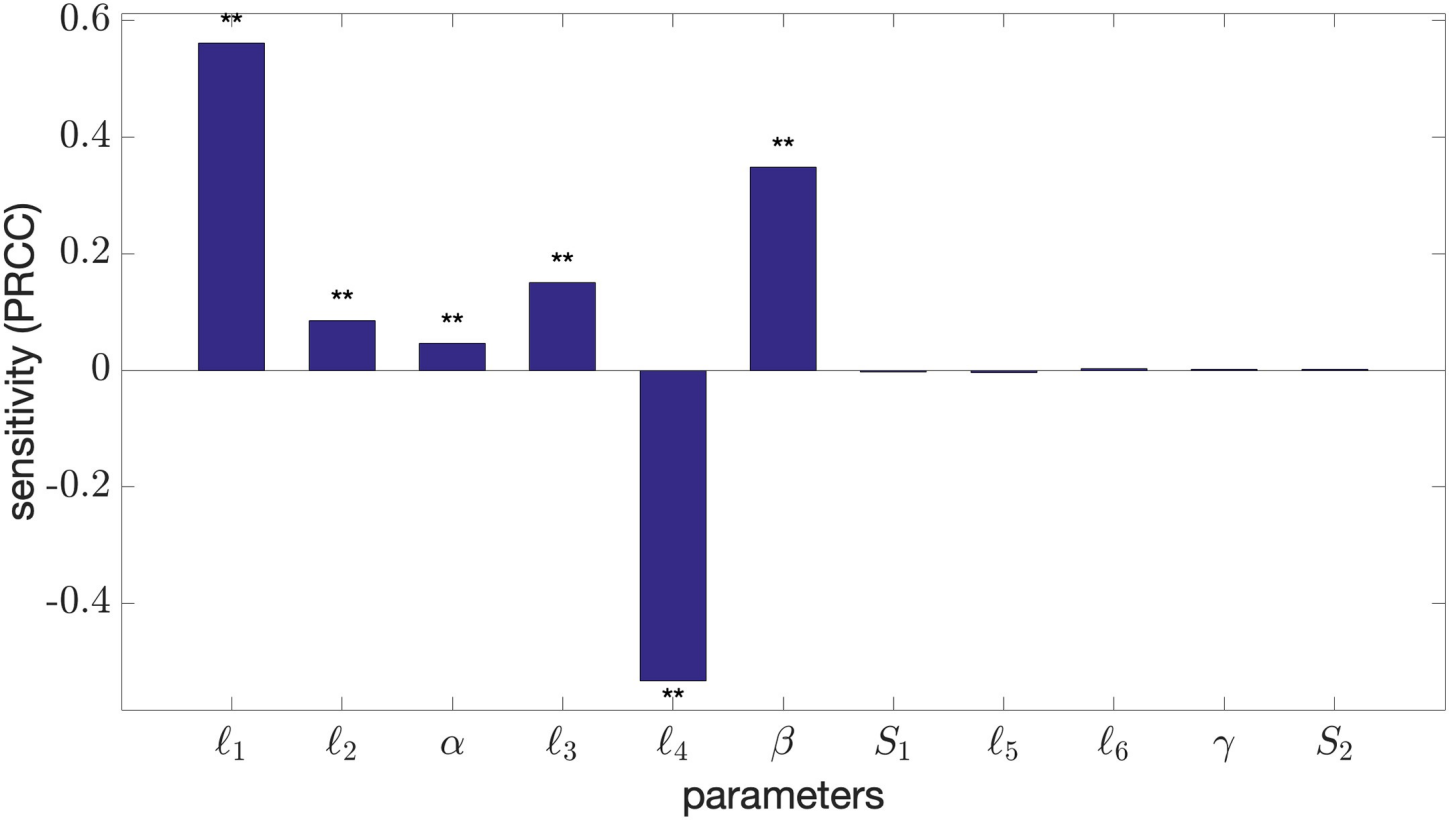
Sensitivity analysis on bistability of core control system. PRCC values of the core control model parameters influencing the bistability of the (*G*, *R*) hysteresis curve. The double asterisk (**) indicates a p-value of less than 0.01. The sample size carried out in the method is *N* = 100, 000.

**Fig 5:**
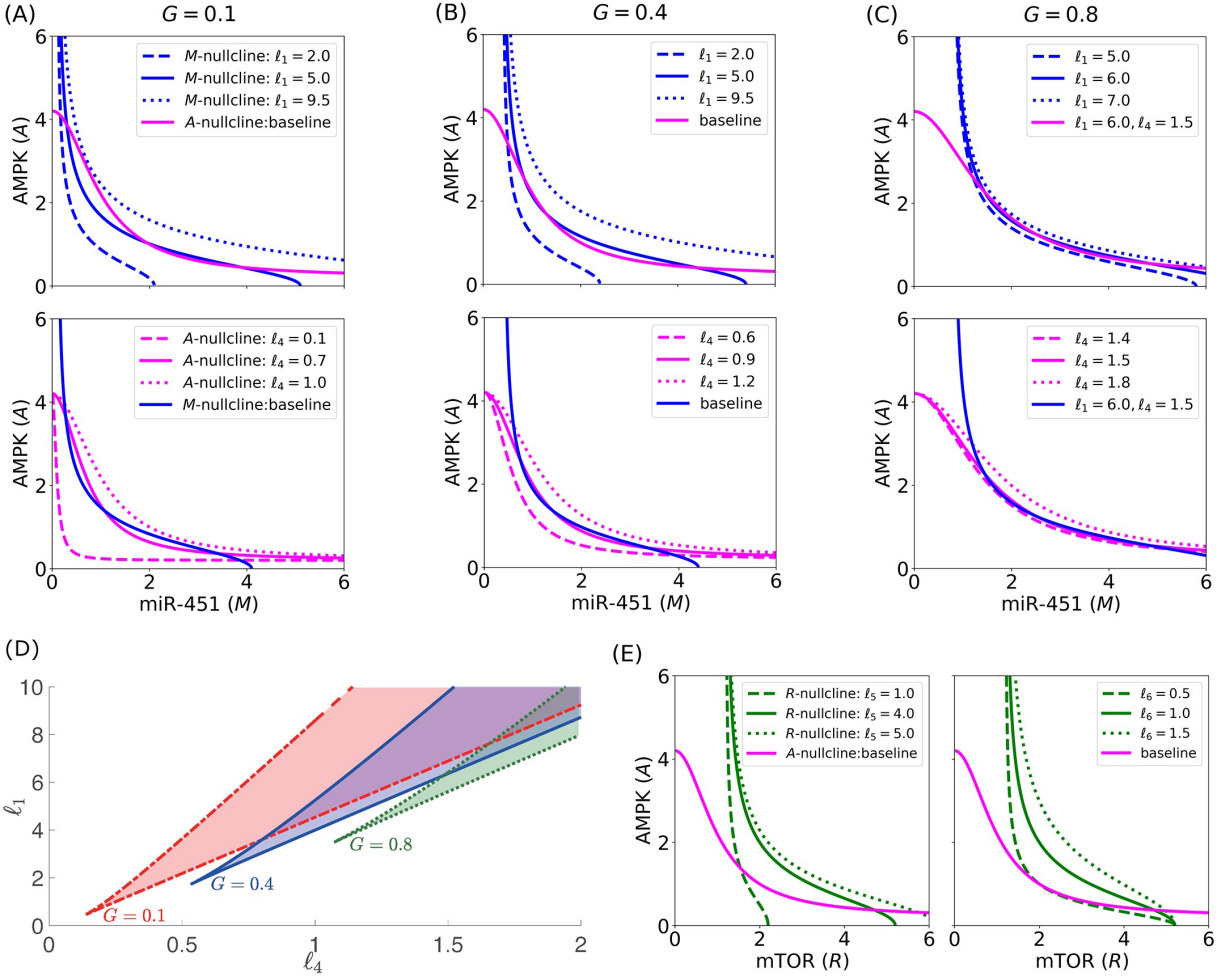
Effect of parameters *ℓ*_1_, *ℓ*_4_, *ℓ*_5_, *ℓ*_6_ on the core control nullclines and bistability. *M*- and *A*-nullclines for different *ℓ*_1_ and *ℓ*_4_ values for specific glucose levels: (A) *G* = 0.1, (B) *G* = 0.4, and (C) *G* = 0.8, showing instances of bistability and monostability. (D) Bifurcation curves for *ℓ*_1_ and *ℓ*_4_ and shaded region of bistability. (E) *R*- and *A*-nullclines for different *ℓ*_5_ and *ℓ*_6_ values showing monostability.

**Fig 6:**
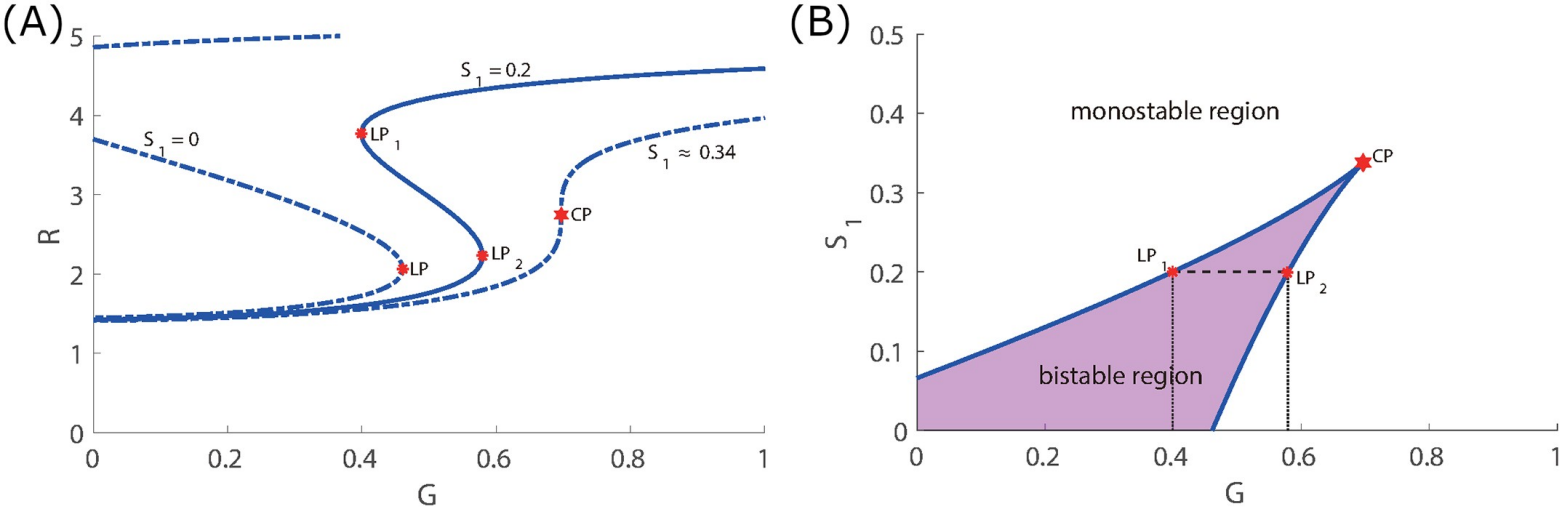
Codimension 2 bifurcation. (A) Hysteresis diagram of mTOR concentration over glucose level for three different values of *S*_1_ = 0, 0.2, 0.34. (B) Codimension 2 bifurcation varying *G* and *S*_1_ showing the equilibrium curves and cusp point (CP). Bistable and monostable regions are also depicted.

In the miR-451-AMPK-mTOR core control system developed by Kim and colleagues [14, 16–18, 40], the bistability regime of main variables emerges in response to glucose levels. As it was shown in [40], the existence and size of the bistability window (|*W*^*b*^|) depend on other essential parameters and may disappear under perturbations of parameters. As it will be shown later (Figs 4 and 5), some of key parameters are sensitive in creation or destroying the bistability while other parameters are not. After achieving the equilibrium, continuation of the curve is computed by varying the glucose concentration *G* and bifurcation points are detected labeled *LP* and *CP* for limit and cusp points, respectively. Fig 6A illustrates the hysteresis diagram (*G*, *R*)−curves for different *S*_1_ values. In order to obtain the cusp point (CP), fold continuation is computed starting at a limit point where two parameters *G* and *S*_1_ are activated resulting to codim 2 bifurcations. Both parameters (*G*, *S*_1_) are varied along the curve where each point is a limit point for the equilibrium curve at the corresponding value of *S*_1_. This is depicted in Fig 6B. The cusp point gives the threshold values *th*_M_, *th*_A_, and *th*_R_. A Matlab software MatCont was used for numerical continuation and bifurcation study of continuous and discrete dynamical systems [46].

The governing model equations for the dimensionless intracellular signaling dynamics are then described by the following ordinary differential equations

$$\begin{array}{c} \begin{array}{cc} \frac{dG}{dt} & =u_{1}\left(t\right)-\mu_{G}G,\\ \frac{dM}{dt} & =G+\frac{\ell_{1}\ell_{2}^{2}}{\ell_{2}^{2}+\alpha A^{2}}-M,\\ \frac{dA}{dt} & =\frac{1}{\epsilon_{1}}(S_{1}+\frac{\ell_{3}\ell_{4}^{2}}{\ell_{4}^{2}+\beta M^{2}}-A),\\ \frac{dR}{dt} & =\frac{1}{\epsilon_{2}}(S_{2}+\frac{\ell_{5}\ell_{6}^{2}}{\ell_{6}^{2}+\zeta \left(D\right)\gamma A^{2}}-R),\\ \frac{dD}{dt} & =u_{2}\left(t\right)-\mu_{D}D,\\ \frac{d[CycB]}{dt} & =k_{1}-\left(k_{2}^{\prime }+k_{2}^{\prime \prime }\left[Cdh1\right]+\left[p27/p21\right]\left[HIF\right]\right)\left[CycB\right],\\ \frac{d[Cdh1]}{dt} & =\frac{\left(k_{3}^{\prime }+k_{3}^{\prime \prime }\left[p55cdc_{A}\right]\right)\left(1-\left[Cdh1\right]\right)}{J_{3}+1-\left[Cdh1\right]}-\frac{k_{4}\left[mass_{s}\right]\left[CycB\right]\left[Cdh1\right]}{J_{4}+\left[Cdh1\right]},\\ \frac{d[p55cdc_{T}]}{dt} & =k_{5}^{\prime }+k_{5}^{\prime \prime }\frac{{\left(\left[CycB\right]\left[mass_{s}\right]\right)}^{n}}{J_{5}^{n}+{\left(\left[CycB\right]\left[mass_{s}\right]\right)}^{n}}-k_{6}\left[p55cdc_{T}\right],\\ \frac{d[p55cdc_{A}]}{dt} & =\frac{k_{7}\left[Plk1\right]\left(\left[p55cdc_{T}\right]-\left[p55cdc_{A}\right]\right)}{J_{7}+\left[p55cdc_{T}\right]-\left[p55cdc_{A}\right]}-\frac{k_{8}\left[Mad\right]\left[p55cdc_{A}\right]}{J_{8}+\left[p55cdc_{A}\right]}-k_{6}\left[p55cdc_{A}\right],\\ \frac{d[Plk1]}{dt} & =k_{9}\left[mass_{s}\right]\left[CycB\right]\left(1-\left[Plk1\right]\right)-k_{10}\left[Plk1\right],\\ \frac{d[mass]}{dt} & =\mu \left[mass\right](1-\frac{\left[mass\right]}{m^{*}}). \end{array} \end{array}$$ (2)

The cell cycle dynamics and the regulatory core control system are linked by a variable called *pseudo-mass* ([*mass*_s_]) given by

$$\begin{array}{c} \left[mass_{s}\right]=\left[mass\right]+\frac{\zeta_{1}(\frac{1}{R}{)}^{n_{1}}}{K_{m}^{n_{1}}+(\frac{1}{R}{)}^{n_{1}}}. \end{array}$$ (3)

The oxygen dynamics through HIF-1 *α* is described as

$$\begin{array}{c} \left[HIF\right]=\frac{\zeta_{2}(\frac{1}{K}{)}^{n_{2}}}{K_{H}^{n_{2}}+(\frac{1}{K}{)}^{n_{2}}}, \end{array}$$ (4)

and the growth rate *μ* is expressed as

$$\begin{array}{c} \mu =\mu^{+}+\varepsilon \hat{\mu }, \end{array}$$ (5)

where $\hat{\mu }$ is the probability density function with uniform distribution between −1 and 1. This growth rate formulation introduces cell cycle heterogeneity of length between 20 and 30 hrs to account for the natural variability between cell growth rates and to have a non-synchronous population [41]. The model parameters in system (2) are listed in Tables 1 and 2.

**Table 1:** Parameters in the core control (miR-451–AMPK–mTOR) model.

Par	Description	Value	Ref
*μ*_*G*_	glucose consumption rate	0.5	[17, 47]
*ℓ*_1_	miR-451 autocatalytic production rate	4.0	[14, 17]
*ℓ*_2_	Hill-type coefficient	1.0	[14, 17]
*α*	inhibition strength of miR-451 by AMPK complex	1.6	[14, 17]
*th*_M_	threshold of miR-451 for invasion/growth switch	1.84	computed*
*ℓ*_3_	AMPK autocatalytic production rate	4.0	[14, 17]
*ℓ*_4_	Hill-type coefficient	1.0	[14, 17]
*β*	inhibition strength of AMPK complex by miR-451	1.0	[14, 17]
*th*_A_	threshold of AMPK for invasion/growth switch	1.25	computed*
*S*_1_	signaling source of AMPK	0.2	[14, 17]
*ϵ*_1_	scaling factor (slow dynamics) of AMPK complex	0.02	[11, 14, 17, 48, 49]
*ℓ*_5_	AMPK autocatalytic production rate	4.0	[14, 17]
*ℓ*_6_	Hill-type coefficient	1.0	[14, 17]
*γ*	inhibition strength of AMPK complex by miR-451	1.0	[14, 17]
*th*_R_	threshold of mTOR for invasion/growth switch	2.76	computed*
*S*_2_	signaling source of mTOR	1.2	[14, 17]
*ϵ*_2_	scaling factor (slow dynamics) of mTOR	0.02	[11, 14, 17, 48, 49]
*μ*_*D*_	drug decay rate	1.316	[41, 50]

*The values are obtained using Matlab numerical bifurcation toolbox Matcont [46].

**Table 2:** Parameters in the cell cycle dynamics model.

Par	Description	Value	Ref
*k*_1_	production rate of [*CycB*]	0.12(*h*^−1^)	[23, 24]
$k_{2}^{\prime }$	degradation rate of [*CycB*]	0.12(*h*^−1^)	[23, 24]
$k_{2}^{\prime \prime }$	degradation rate of [*CycB*] by [*Cdh*1]	4.5(*h*^−1^)	[23, 24]
[p27/p21]	inhibitory effect of p21 or p27 genes	1.05	[41]
*K*	oxygen concentration threshold	0.01	[41]
[CycB]_th_	threshold for cell division	0.1	[23, 24]
$k_{3}^{\prime }$	activation rate of [*Cdh*1]	3.0(*h*^−1^)	[23, 24]
$k_{3}^{\prime \prime }$	activation rate of [*Cdh*1] by [*p*55*cdc*_*A*_]	30(*h*^−1^)	[23, 24]
*k*_4_	inactivation rate of [*Cdh*1] by [*CycB*]	105(*h*^−1^)	[23, 24]
*J*_3_	Michaelis-Menten activation constant	0.04	[23, 24]
*J*_4_	Michaelis-Menten inactivation constant	0.04	[23, 24]
$k_{5}^{\prime }$	production rate of [*p*55*cdc*_*T*_]	0.015(*h*^−1^)	[23, 24]
$k_{5}^{\prime \prime }$	transcription rate of [*p*55*cdc*_*T*_] by [*CycB*]	0.6(*h*^−1^)	[23, 24]
*k*_6_	degradation rate of [*p*55*cdc*_*T*_]	0.3(*h*^−1^)	[23, 24]
*J*_5_	dissociation constant of [*p*55*cdc*_*T*_]	0.3	[23, 24]
*n*	Hill coefficient	4.0	[23, 24]
*k*_7_	activation rate of [*p*55*cdc*_*A*_] by [*Plk*1]	3.0(*h*^−1^)	[23, 24]
*k*_8_	inactivation rate of [*p*55*cdc*_*A*_] by [*Mad*]	1.5(*h*^−1^)	[23, 24]
*J*_7_	Michaelis-Menten activation constant	0.001	[23, 24]
*J*_8_	Michaelis-Menten inactivation constant	0.001	[23, 24]
[*Mad*]	spindle checkpoint genes concentration	1.0	[23, 24]
*k*_9_	activation rate of [*Plk*1] by [*CycB*]	0.3(*h*^−1^)	[23, 24]
*k*_10_	degradation rate of [*Plk*1]	0.06(*h*^−1^)	[23, 24]
*μ*^+^	specific growth rate	0.03	[23, 24]
m*	maximum size to which a cell may grow	10	[23, 24]
*ε*	cell cycle heterogeneity growth rate parameter	0.006	[41]
*ζ*_1_	Hill-type parameter in [*mass*_*s*_]	2.5	[17]
*n*_1_	Hill-type parameter in [*mass*_*s*_]	10	[17]
*K*_*m*_	Hill-type parameter in [*mass*_*s*_]	0.5	[17]
*ζ*_2_	Hill-type parameter in [*HIF*]	1.0	[41]
*n*_2_	Hill-type parameter in [*HIF*]	10.0	[41]
*K*_*H*_	Hill-type parameter in [*HIF*]	10.0	[41]
*μ*_*D*_	Decay rate of drug	1.316	[41, 50]

**Fig 7:**
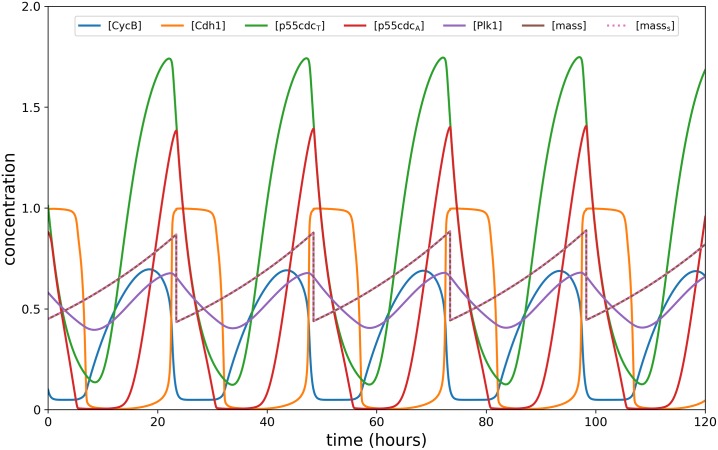
Typical cell cycle dynamics. Dynamics of intracellular proteins, mass, and mass_s_ of the cell cycle model in response to constant (intermediate) high glucose level.

In the current model formulation, mTOR (*R*) is activated/inactivated in the same way as miR-451 (*M*). In addition, *R* links the core control system and the cell cycle dynamics via the *pseudo-mass* ([*mass*_s_]) which influences the cell mass [*mass*] and the intracellular proteins (refer to Eq (3)). Therefore, when glucose supply is high, *R* is up-regulated (proliferative phase; up-regulated *M*, down-regulated *A*) and [*mass*_s_] ≈ [*mass*] yielding a typical cell cycle (see Fig 7). On the other hand, when glucose supply is low, *R* is down-regulated (migratory phase; down-regulated *M*; up-regulated *A*) and [*mass*_s_] exceeds [*mass*] influencing the cells to enter into resting phase G0 [40].

Let us assume that glucose (*G*) and drug (*D*) can be regulated through intravenous infusions and can be periodically administered. For illustrative purposes, consider 3h infusions every 12h with maximum dosage of 1 unit, and refer this as *regular infusion*. We then have

$$\begin{array}{c} u_{j}\left(t\right)=\{\begin{array}{c} 1\;\text{for}\;t\in [12n,12n+3],\;n=0,1,2,\ldots \\ 0\;\text{otherwise.} \end{array}\;\text{for}\;j=G,D. \end{array}$$ (6)

**Fig 8:**
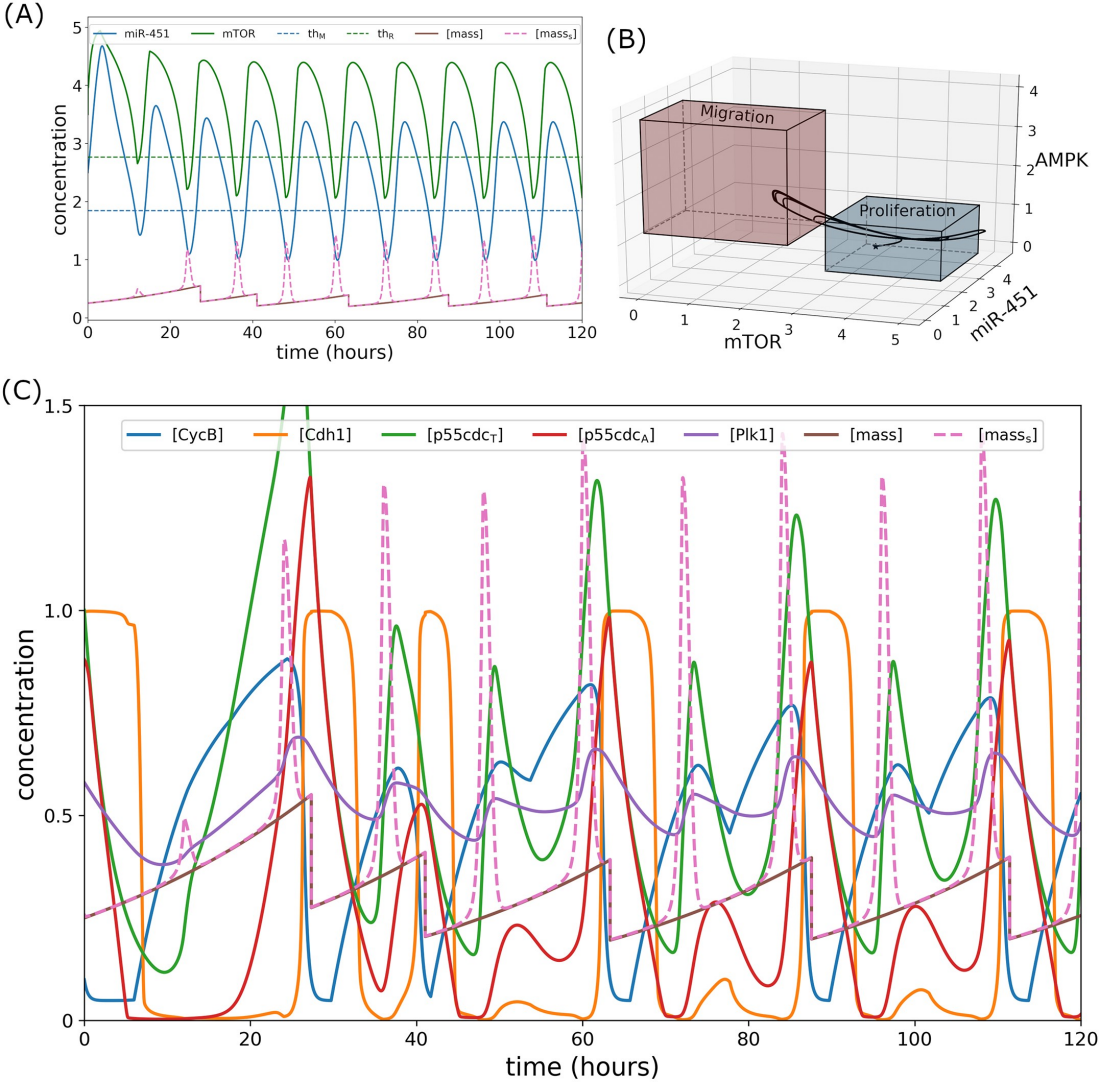
Intracellular dynamics under regular glucose and drug infusions. (A) Concentration profiles of miR-451 (*M*) and mTOR (*R*) fluctuating around the threshold values under regular infusions. Peaks in pseudo-mass are generated when *M* and *R* crosses *th*_M_ and *th*_R_, respectively. (B) Trajectory of mTOR–miR-451–AMPK concentration profiles switching between proliferation and migration mode. (C) Dynamics of intracellular proteins, mass, and mass_s_ of the cell cycle model in response to regular glucose and drug infusions.

The intracellular dynamics under regular infusion can be seen in Fig 8. Since glucose and drug levels oscillate, it follows that *M* and *R* also periodically fluctuates around the threshold (*th*_M_ ≈ 1.87, *th*_R_ ≈ 2.76) as can be seen in Fig 8A. Note that when *M* and *R* crosses the threshold value from above, respectively, peak in pseudo-mass is generated. It can thus be inferred that these peaks indicate cell migration since *M* and *R* levels are below their respective threshold values. As shown in Fig 8B, a trajectory of core control concentrations in mTOR-miR-451–AMPK space switches between proliferation and migration region. A closer look at the intracellular cell cycle proteins, mass, and mass_s_ dynamics are illustrated in Fig 8C. Note that regular infusion significantly perturb the cell cycle dynamics stimulating several cell divisions (see mass profiles) and cell migration (see mass_s_). Indeed, fluctuating glucose levels in the microenvironment leads to the dichotomy of grow and go dynamics of glioblastoma cells yielding bigger tumor mass as reported in [14], even in the presence of (fluctuating) drug concentrations to keep the cells in proliferation phase.

### Optimal control problem

In the current investigation, we aim to regulate the amount of glucose and drug infusions to up-regulate miR-451 and mTOR above its threshold values inducing cell proliferation avoiding migration to neighboring tissues. The modeling approach utilizes optimal control theory to identify infusion administration protocols. Let *u*_*G*_(*t*) and *u*_*D*_(*t*) be the controls of the system representing dose rates of glucose and drug intravenous administrations, respectively. Two different administration protocols are examined, namely, (1) *concomitant*, and (2) *alternating* glucose and drug infusions, with the following objective functionals

$$\begin{array}{c} \begin{array}{cc} J_{\text{con}}(u_{G}\left(t\right),u_{D}\left(t\right)) & =\int_{t_{0}}^{t_{1}}(M\left(t\right)+R\left(t\right)-(\frac{B_{1}}{2}u_{G}{\left(t\right)}^{2}+\frac{B_{2}}{2}u_{D}{\left(t\right)}^{2}))\;dt,\\ J_{\text{alt}}(u_{1}\left(t\right),u_{2}\left(t\right)) & =\int_{t_{0}}^{t_{1}}(M\left(t\right)+R\left(t\right)-\frac{B_{1}}{2}u_{G}{\left(t\right)}^{2})\;dt\\ & \;\;\;+\int_{t_{1}}^{t_{2}}(R\left(t\right)-\frac{B_{1}}{2}u_{D}{\left(t\right)}^{2})\;dt, \end{array} \end{array}$$ (7)

respectively. Here, *M*(*t*) and *R*(*t*) denote the level of miR-451 and mTOR concentrations, respectively. Parameters *B*_1_ and *B*_2_ are weight factors measuring the relative cost based on maximizing miR-451 *M*(*t*) and mTOR *R*(*t*), and administering glucose and drug intravenous infusions over a specified time interval, respectively. The control costs are modeled by the linear combination of quadratic terms $u_{G}^{2}\left(t\right)$ and $u_{D}^{2}\left(t\right)$. Our objective is to find optimal infusion regimen for glucose and drug administrations, denoted by $u_{G}^{*}\left(t\right)$ and $u_{D}^{*}\left(t\right)$, such that the objective functionals are satisfied, that is,

$$\begin{array}{c} \begin{array}{cc} J_{\text{con}}(u_{G}^{*}\left(t\right),u_{D}^{*}\left(t\right)) & =\underset{\Omega_{\text{con}}}{\text{max}}J_{\text{con}}(u_{G}\left(t\right),u_{D}\left(t\right)),\\ J_{\text{alt}}(u_{G}^{*}\left(t\right),u_{D}^{*}\left(t\right)) & =\underset{\Omega_{\text{alt}}}{\text{max}}J_{\text{alt}}(u_{G}\left(t\right),u_{D}\left(t\right)), \end{array} \end{array}$$ (8)

where

$$\begin{array}{c} \begin{array}{cc} \Omega_{\text{con}} & =\{u_{G}\left(t\right),u_{D}\left(t\right)\in ℒ\left(t_{0},t_{1}\right)|0\leq u_{G}\left(t\right),u_{D}\left(t\right)\leq u_{\text{max}},t\in [t_{0},t_{1}]\},\\ \Omega_{\text{alt}} & =\{u_{G}\left(t\right)\in ℒ\left(t_{0},t_{1}\right),u_{D}\left(t\right)\in ℒ\left(t_{1},t_{2}\right)|\\ & \;0\leq u_{G}\left(t\right)\leq u_{\text{max}},t\in [t_{0},t_{1}],0\leq u_{D}\left(t\right)\leq u_{\text{max}},t\in [t_{1},t_{2}]\}, \end{array} \end{array}$$ (9)

The bounds for the controls represent the limits on dose rates for glucose and drug administrations. Assuming that miR-451, AMPK, and mTOR responses are regulated by glucose levels and influenced by drug levels, our control strategies deal with finding optimal control regimens for both glucose and drug intravenous infusions. We also note that the existence of optimal controls is guaranteed by standard results in control theory [51]. In this maximization problem, the necessary convexity of the integrand in the objective functional holds. Therefore, we can proceed with applying Pontryagin’s Maximum Principle [52]. An iterative method is used for solving the optimality system which is a two-point boundary value problem having initial conditions for the state variables and terminal conditions for the adjoints. Numerical simulations are obtained using a fourth-order iterative Runge-Kutta method. Given the initial conditions and guess for the controls, state equations are solved using the forward scheme while the corresponding adjoint equations are solved using the backward scheme with the transversality conditions. The controls are updated by using a convex combination of the previous controls and the value from the characterizations. This is commonly referred as the *Forward–Backward Sweep Method (FBSM)* which is shown to be convergent [53]. Further details on the optimal control under study can be found in the S1 Appendix.

## Results and discussion

### Sensitivity analysis on bistability of the miR-451-AMPK-mTOR system

In Kim *et al*. [40] (see Fig S5 in Supplementary Appendix File in [40]), sensitivity analysis of the core control miR-451-AMPK-mTOR model (equations for *M*, *A*, *R* in Eq (2)) was performed in order to determine which parameters have the most/least influence on the reference output (main variables). All the core control parameters were considered to assess their corresponding influence on the miR-451, AMPK, and mTOR activity. It was inferred that miR-451 activity will be enhanced by increasing glucose signal (*G*) and autocatalytic production rates (*ℓ*_1_, *ℓ*_2_) of miR-451. These parameters were negatively correlated with AMPK activity due to the mutual antagonistic mechanism between miR-451 and AMPK complex. In addition, AMPK activity will be up-regulated by an increase in signaling source *S*_1_ and autocatalytic rates (*ℓ*_3_, *ℓ*_4_) of the AMPK complex in the model. It has been also noted that increasing *S*_1_ and the inhibition strength of mTOR by AMPK (*γ*) down-regulates mTOR level. In a similar fashion, *S*_2_ is strongly positively correlated with mTOR levels but little correlated with *G*, *ℓ*_1_, *ℓ*_2_, *ℓ*_3_, *ℓ*_4_, *α*, *β*, *ϵ*_1_, *ϵ*_2_ at time 100.

In this work sensitivity analysis is carried out to determine which model parameters have consequential effect in achieving or inhibiting bistability in the (*G*, *R*)−curve. As in [40], a method from [54] is adapted where a range for each parameter is selected and divided into *N* intervals of uniform length. For each parameter of interest, a partial rank correlation coefficient (PRCC) value is computed. In order to obtain the PRCC values, *Latin Hypercube Sampling* (LHS), a stratified sampling without replacement technique, is chosen. The PRCC values range between –1 and 1 with the sign determining whether a change in the parameter value will promote (+) or suppress (−) bistability. The following algorithm is performed:

1. Assign a probability distribution $D[\mu_{j,min},\mu_{j,max}]$ to each parameter *μ*_*j*_ and let *N* be the number of samples to be selected. Divide the interval [*μ*_*j*,*min*_, *μ*_*j*,*max*_] into *N* equiprobable subintervals, and draw an independent sample from each subinterval.
2. Assemble the LHS matrix *L*, wherein each row of *L* represents a unique combination of parameters sampled without replacement.
3. For each row of the LHS matrix *L*, solve for mTOR *R* in terms of glucose *G* and check for bistability window *W*^*b*^. If bistability exists assign 1 to output variable *W*^*b*^ in matrix *Y*, else assign −1.
4. Rank-transform the matrices *L* and *Y* to obtain *L*_*R*_ and *Y*_*R*_. By rank-transform, we mean to replace the value by its rank when the data are sorted from lowest to highest, e.g. the smallest value is assigned a rank 1. Tied values are assigned an average rank.
5. Fix a parameter *μ*_*j*_, which is encoded in the *j*^*th*^ column in the matrix *L*_*R*_. Form the following linear regression models using the data matrices *L*_*R*_ and *Y*_*R*_ for *μ*_*j*_ and *y*, respectively:
  $$\begin{array}{ccc} {\hat{\mu }}_{j} & = & c_{0}+\sum_{p=1,p\neq j}^{n_{P}}c_{p}\mu_{p}, \end{array}$$ (10)
  $$\begin{array}{ccc} \hat{y} & = & b_{0}+\sum_{p=1,p\neq j}^{n_{P}}b_{p}\mu_{p}. \end{array}$$ (11)
  Compute $\left(\mu_{j}-{\hat{\mu }}_{j}\right)$ and $\left(y-\hat{y}\right)$, the residuals in the input parameter and the output after removing the linear effects of the other input parameters.
6. Obtain the PRCC of *μ*_*j*_ using
  $$\begin{array}{c} PRCC_{\mu_{j},y}=\frac{\text{Cov}(\mu_{j}-{\hat{\mu }}_{j},y-\hat{y})}{\sqrt{\text{Var}\left(\mu_{j}-{\hat{\mu }}_{j}\right)\text{Var}\left(y-\hat{y}\right)}}. \end{array}$$ (12)
7. Repeat Steps 5 and 6 for the remaining parameters.

Fig 4 shows the sensitivity (PRCC value) of the model parameters in promoting (+) or destroying (−) bistability. It illustrates that a change in the miR-451 autocatalytic production rate (*ℓ*_1_) or inhibition strength of AMPK complex by miR-451 (*β*) enhances bistability. On the contrary, the Hill-type coefficient *ℓ*_4_ in the regulation of AMPK is responsible for losing bistability. It should be noted that due to the model’s structure, *ℓ*_1_ up-regulates miR-451 which in turn increases mTOR and suppress AMPK activity. The parameter *ℓ*_4_ promotes AMPK complex and down-regulates miR-451 and mTOR. However, other parameters *ℓ*_2_, *α*, *ℓ*_3_, *S*_1_, *ℓ*_5_, *ℓ*_6_, *γ*, *S*_2_ are little sensitive in emergence or destruction of the bistability.

The bistability of miR-451–AMPK–mTOR core control system depends on the geometric structure of its nullclines. In particular, bistability arises when *M*- and *A*-nullclines (i.e., $\frac{dM}{dt}=0$ and $\frac{dA}{dt}=0$) intersect at three distinct points, producing one unstable and two stable steady states. The nullclines intersect three times due to their sigmoidicity influenced by catalytic rates *ℓ*_1_ and *ℓ*_4_. These rates must be proportionate for a given glucose *G* level. Otherwise, the nullclines will intersect only once. This bistability condition has been shown for a genetic toggle switch in *E. coli* [42]. Fig 5A–5C depict the *M*- and *A*-nullclines under different *G* levels with various *ℓ*_1_ and *ℓ*_4_ values. The bifurcation curves for *ℓ*_1_, *ℓ*_4_ and region of bistability for specific *G* levels are depicted in Fig 5D. Under given circumstances, *ℓ*_1_ and *ℓ*_4_ showed significant sensitivities in the bistability of the system. On the contrary, both *ℓ*_5_ and *ℓ*_6_ affect the *R*-nullcline only. It is illustrated in Fig 5E that for several *ℓ*_5_ and *ℓ*_6_ values, *R*- and *A*-nullclines intersect at only one point, leading to a single steady state. In effect, *M*- and *A*-nullclines will also intersect once, producing monostability. Hence, *ℓ*_5_ and *ℓ*_6_ show insignificant consequence in achieving bistability (see Fig 4).

In the following numerical experiments, it is assumed that initially glioma cells are in growth phase (a probable occurrence of post primary tumour surgery) with *M* > *th*_M_, *A* < *th*_A_, and *R* > *th*_R_. It is also considered that glucose and drug can be administered exogenously as intravenous infusions. Further, the weight parameters *B*_1_ = 1 and *B*_2_ = 1 are used as default values unless specified.

### Concomitant infusion control

**Fig 9:**
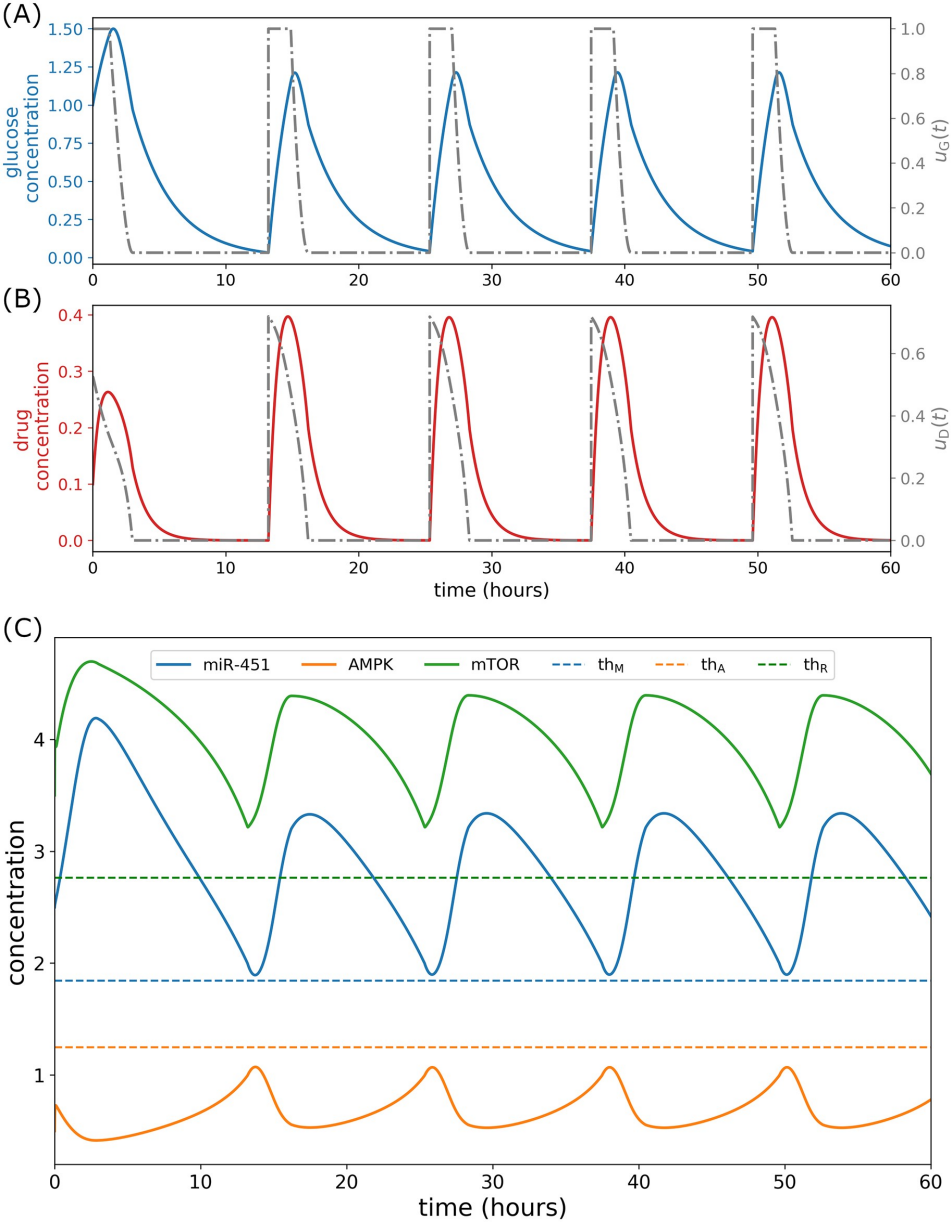
Concomitant glucose and drug control. (A) Glucose control and concentration levels, (B) drug control and concentration levels, and (C) concentration profiles of miR-451, AMPK complex, and mTOR under concomitant control.

**Fig 10:**
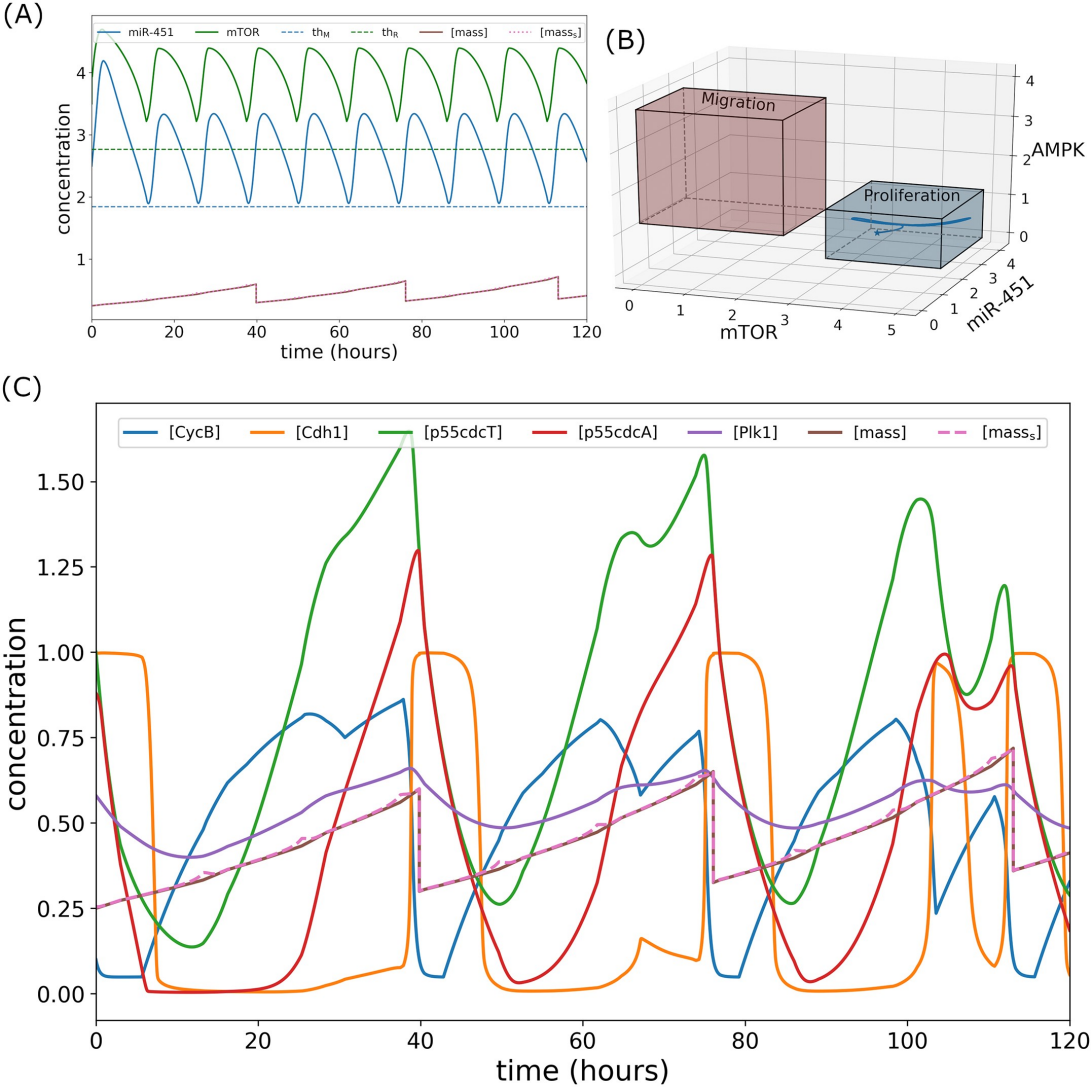
Intracellular dynamics under concomitant glucose and drug control. (A) Concentration profiles of miR-451 (*M*) and mTOR (*R*) above the threshold values under concomitant infusions. (B) Trajectory of mTOR–miR-451–AMPK concentration profiles restrained in the proliferation region. (C) Dynamics of intracellular proteins, mass, and mass_s_ of the cell cycle model in response to concomitant glucose and drug infusions.

In this strategy, glucose and drug infusions are administered simultaneously, in particular, both controls *u*_*G*_(*t*) and *u*_*D*_(*t*) are infused at the same. Thus, this is referred to as *concomitant control*. Here, it is assumed that the drug in consideration which blocks the inhibitory pathway of mTOR by AMPK complex had negligible side effects and had inconsequential chemical reactions with glucose. In order to determine an efficient strategy of concomitant infusion protocol, glucose and drug are administered concurrently at initial time *t* = 0 for 3 hours using the numerical scheme FBSM. Infusion spontaneously increases glucose and drug concentrations as depicted in Fig 9(A) and 9(B). Consequently, miR-451 and mTOR levels are up-regulated while AMPK complex is down-regulated as shown in Fig 9C. A closer look at the control curves shows that both *u*_*G*_(*t*) and *u*_*D*_(*t*) decrease from 0 < *t*_*i*_ < 3 suggesting that both glucose and drug dose rates should be decreased from time *t*_*i*_. This leads to the decrease of glucose and drug concentrations due to consumption and decay. Accordingly, miR-451 and mTOR levels decrease while AMPK complex increases. Before miR-451 crosses the threshold value *th*_M_, FBSM is again applied for the next 3 hours. This suggests a time for the next concomitant administrations in which miR-451 profiles are monitored subsequently. The procedures in tracking miR-451 profiles and applying FBSM for 3 hours are repeated over a specified time duration. Thus, the number of concomitant administrations are determined. It is important to note that keeping miR-451 level above its threshold value consequently confine AMPK and mTOR levels, below and above its corresponding threshold values, respectively (see Fig 9C). Fig 10 depicts the intracellular dynamics under concomitant glucose and drug control. In Fig 10A, it can be observed that both miR-451 and mTOR levels are above their threshold values, and [*mass*_s_] ≈ [*mass*]. Under concomitant control, the trajectory of mTOR–miR-451–AMPK concentration profiles are restricted only in the proliferation region as shown in Fig 10B. The time courses of cell cycle variables under concomitant control is illustrated in Fig 10C. It can be seen that concomitant control induces fewer (only 3) mass divisions over 120h period as compared to regular infusions with 5 mass divisions, see Fig 8C. It should be noted as well that in concomitant control, mass concentration is increased before division.

### Alternating infusion control

**Fig 11:**
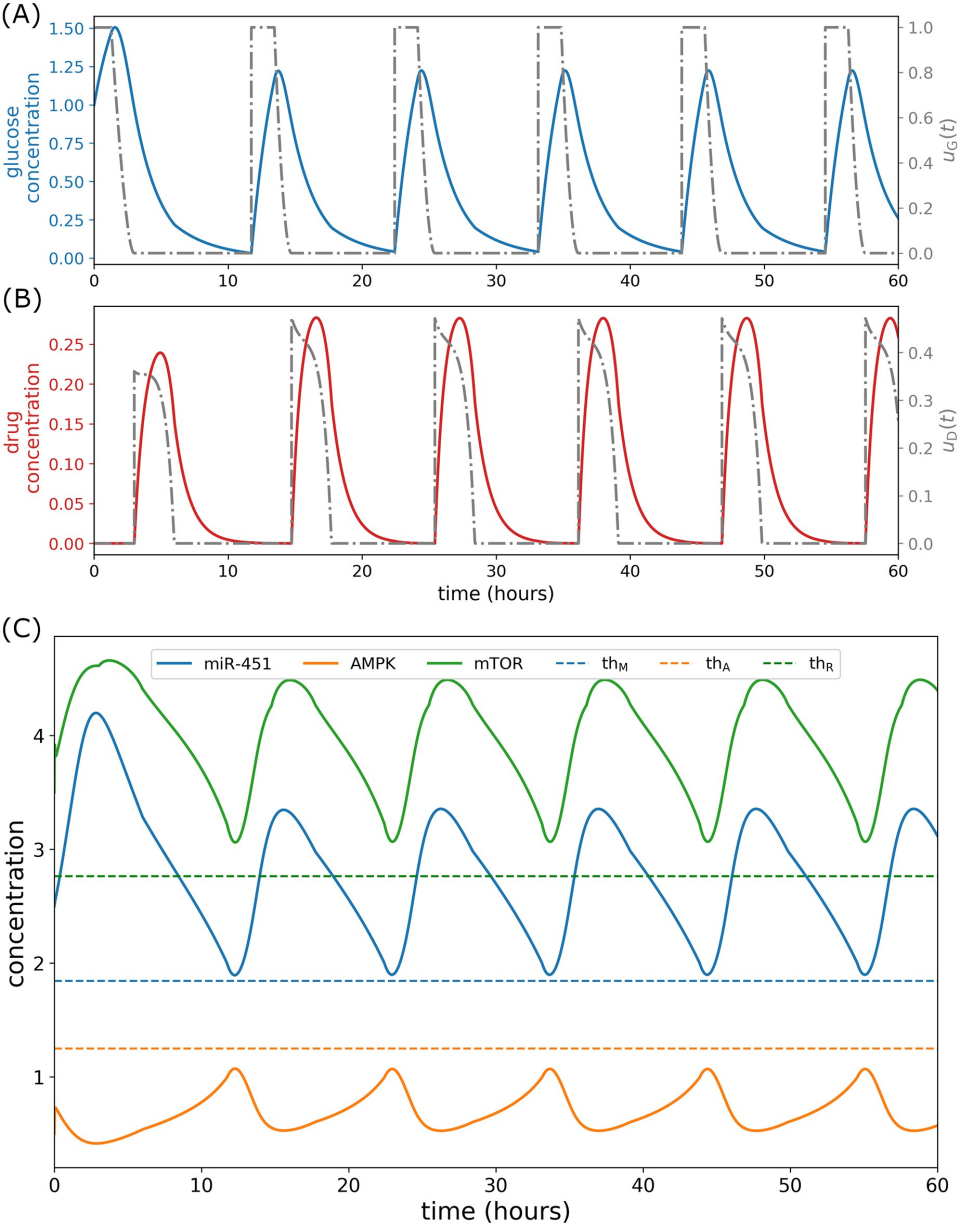
Alternating glucose and drug control. (A) Glucose control and concentration levels, (B) drug control and concentration levels, and (C) concentration profiles of miR-451, AMPK complex, and mTOR under alternating control.

**Fig 12:**
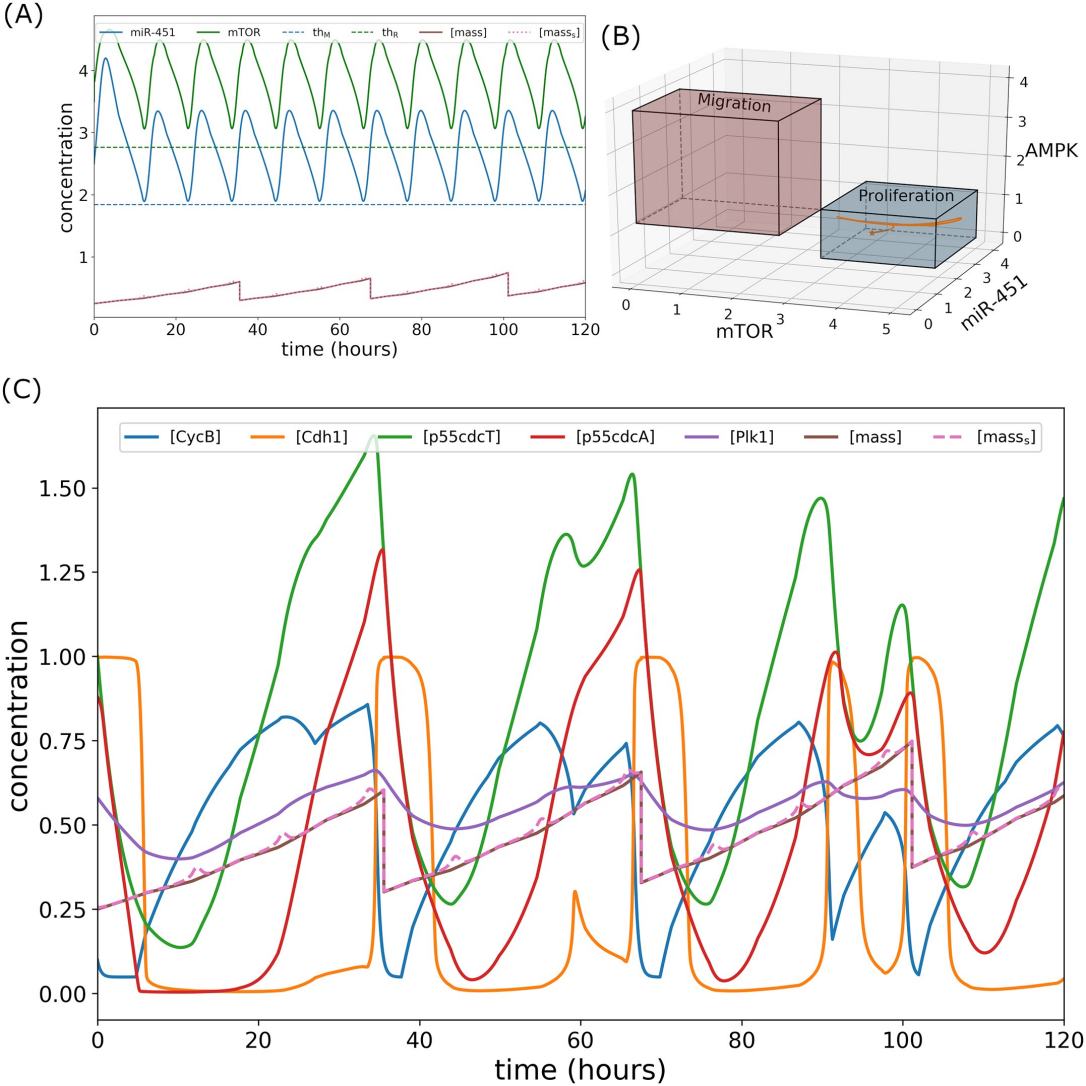
Intracellular dynamics under alternating glucose and drug control. (A) Concentration profiles of miR-451 (*M*) and mTOR (*R*) above the threshold values under concomitant infusions. (B) Trajectory of mTOR–miR-451–AMPK concentration profiles restrained in the proliferation region. (C) Dynamics of intracellular proteins, mass, and mass_s_ of the cell cycle model in response to alternating glucose and drug infusions.

Suppose that concurrent glucose and drug administrations is not plausible due to unwanted chemical reactions. We propose another control strategy that administers glucose *u*_*G*_(*t*) and drug *u*_*D*_(*t*) infusions alternately. At initial time *t* = 0, glucose infusion is obtained by solving the optimal control problem using the numerical scheme FBSM for 3 hours. Next, drug infusion is attained for the next 3 hours in a similar manner. Hence, the controls *u*_*G*_(*t*) and *u*_*D*_(*t*) are applied one after the other. Subsequently, miR-451 levels are then tracked and before it crosses the threshold value, glucose infusion *u*_*G*_(*t*) and drug infusion *u*_*D*_(*t*) are again administered alternately in a similar fashion above, over the specified duration of administration. Thus, the time for the next alternating infusion is determined by tracking miR-451 levels. This strategy is referred simply as *alternating control*. This infusion protocol is shown in Fig 11(A) and 11(B). Note that glucose infusion increases miR-451 levels and drug infusion activates mTOR activities keeping AMPK down-regulated as depicted in Fig 11C. The intracellular dynamics under alternating control is illustrated in Fig 12. As shown in Fig 12A, miR-451 and mTOR levels stay above the threshold values, and [*mass*_s_] ≈ [*mass*]. Fig 12B depicts that mTOR–miR-451–AMPK trajectory are confined in proliferation region. Fig 12C exhibits the time courses of cell cycle variables under alternating control.

### Comparison between control strategies

Both concomitant and alternating control strategies are able to sustain elevated miR-451 and mTOR levels above their threshold values and AMPK levels below its threshold value. It was also shown that mTOR–miR-451–AMPK trajectory is restrained in the proliferation region prohibiting cell migration. Further, number of cell divisions is reduced with slightly higher mass concentration before division compared to regular infusions but lower compared to constant (intermediate) high glucose concentrations. In this section, we compare the cost efficiency of the proposed strategies in terms of frequency of administration, dose per infusion, total glucose and drug amount, relative cost per infusion, and total cost incurred in the administrations. For the following results, the time for simulation duration is considered to be 168 hours (7days).

**Fig 13:**
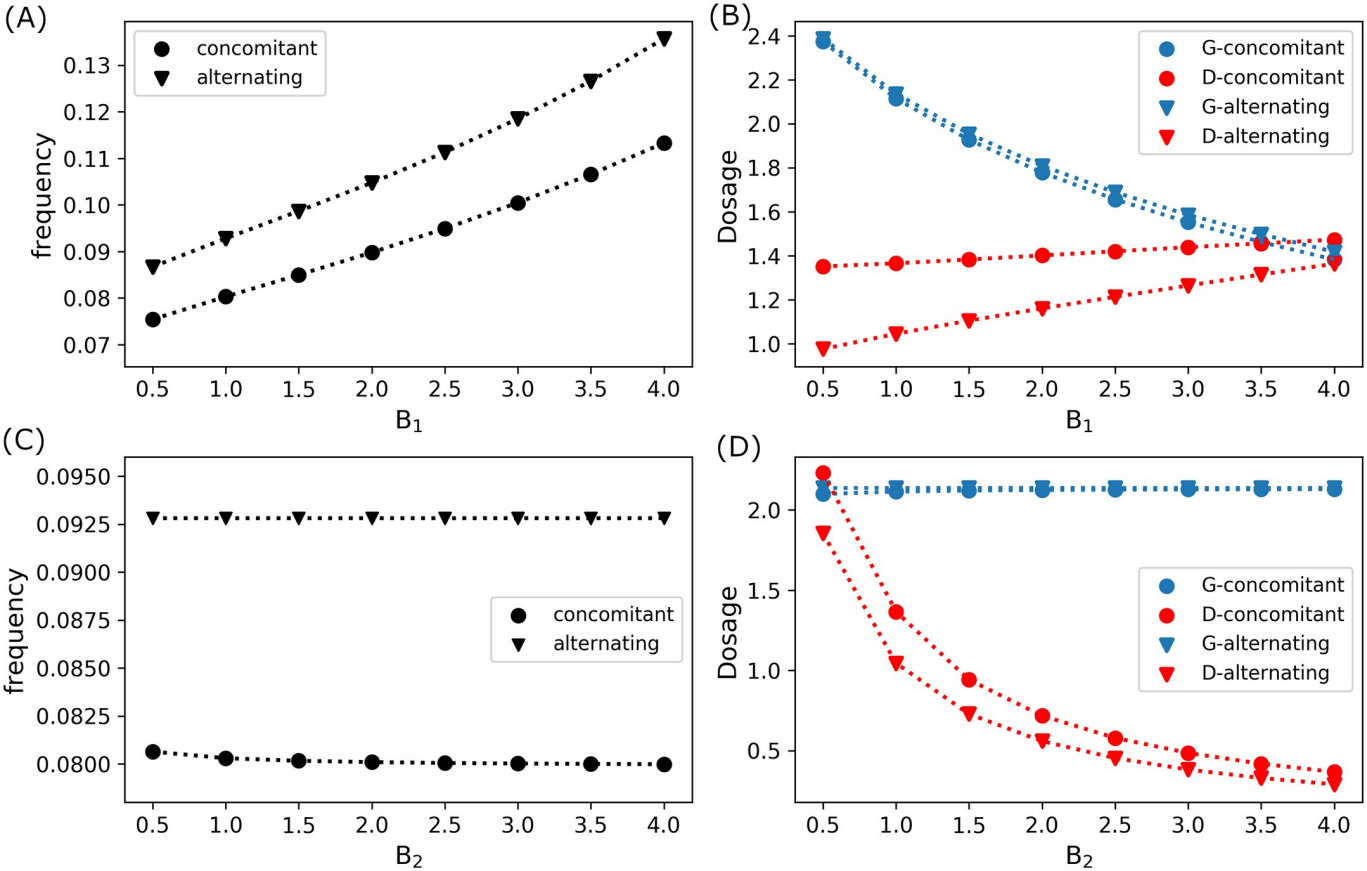
Frequency and dosage of optimal infusions. (A) Frequency and (B) dose per optimal infusion of concomitant (circle) and alternating (triangle) controls with fixed drug administration cost *B*_2_ = 1.0 and varying glucose administration cost *B*_1_. (C) Frequency and (D) dose per optimal infusion of concomitant (circle) and alternating (triangle) controls with fixed glucose administration cost *B*_1_ = 1.0 and varying drug administration cost *B*_2_.

Recall that parameters *B*_1_ and *B*_2_ are the weight factors associated in our objective functional which represent the measure of costs involved in the administration of glucose *u*_*G*_(*t*) and drug *u*_*D*_(*t*) infusions, respectively, which also includes dosage, type, brand, medical fee for administration, etc. Fig 13A shows that as the cost of glucose administration becomes expensive (increasing *B*_1_ values) with fixed drug administration cost (*B*_2_ = 1.0), frequency of concomitant and alternating infusion increases. However, it should be observed that as frequency of administration increases, the optimal glucose dose per infusion decreases with drug dose per infusion increases, see Fig 13B. Increasing drug dosage compensates the decreasing glucose dosage in order to keep mTOR activities up-regulated leading to cell proliferation. On the contrary, if drug administration cost increases (increasing *B*_2_ values) with fixed glucose administration cost (*B*_1_ = 1.0), frequency of administration remains almost constant. This is depicted in Fig 13C. The glucose dose per infusion is almost the same but the drug dose per infusion decreases with increasing *B*_2_ as illustrated in Fig 13D. Further, note that frequency of concomitant infusion control is always lower than that of alternating infusion control. In addition, drug (glucose) dose per infusion of concomitant control is always more (slightly less) than that of alternating control.

**Fig 14:**
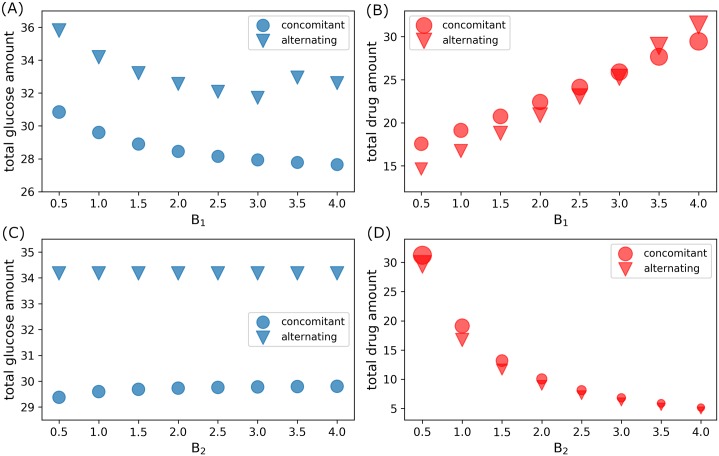
Total glucose and drug amount used in concomitant and alternating control infusions. (A) Total glucose and (B) drug amount used in concomitant (circle) and alternating (triangle) controls with fixed drug administration cost *B*_2_ = 1.0 and varying glucose administration cost *B*_1_. (C) Total glucose and (D) drug amount used in concomitant (circle) and alternating (triangle) controls with fixed glucose administration cost *B*_1_ = 1.0 and varying drug administration cost *B*_2_.

For increasing glucose administration cost (increasing *B*_1_) with fixed drug administration cost (*B*_2_ = 1.0), total amount of glucose used for both control strategies generally decrease (except for high *B*_1_ > 3.0 values), as depicted in Fig 14A, while total amount of drug increases, see Fig 14B. On the other hand, when drug administration becomes expensive (increasing *B*_2_ values) with fixed glucose administration cost (*B*_1_ = 1.0), the total amount of glucose dosage is almost constant (just slightly increasing for concomitant control) as shown in Fig 14C, but the total drug amount is decreasing as can be seen in Fig 14D. As illustrated in Fig 14, concomitant control use less total amount of glucose than that of alternating control. Contrarily, concomitant administration consumes more total drug amount as compared to that of alternating control (except possibly for high glucose administration cost, *B*_1_ > 3.0, see Fig 14B).

**Fig 15:**
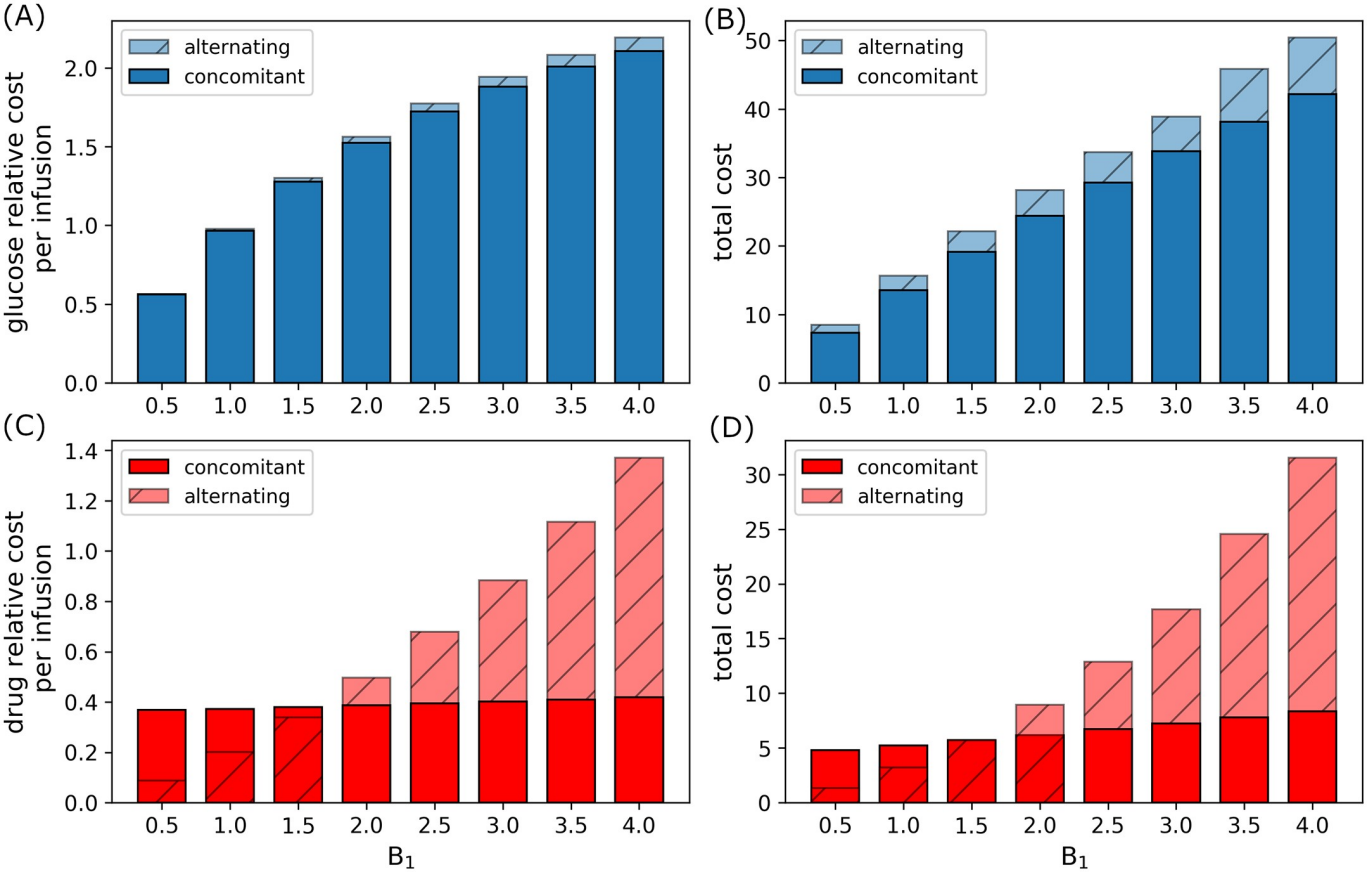
Relative administration cost for varying *B*_1_. Relative glucose administration (A) cost per infusion and (B) total cost, and relative drug administration (C) cost per infusion and (D) total cost incurred for a period of 168*h* (7*d*) for concomitant and alternating controls with increasing *B*_1_.

**Fig 16:**
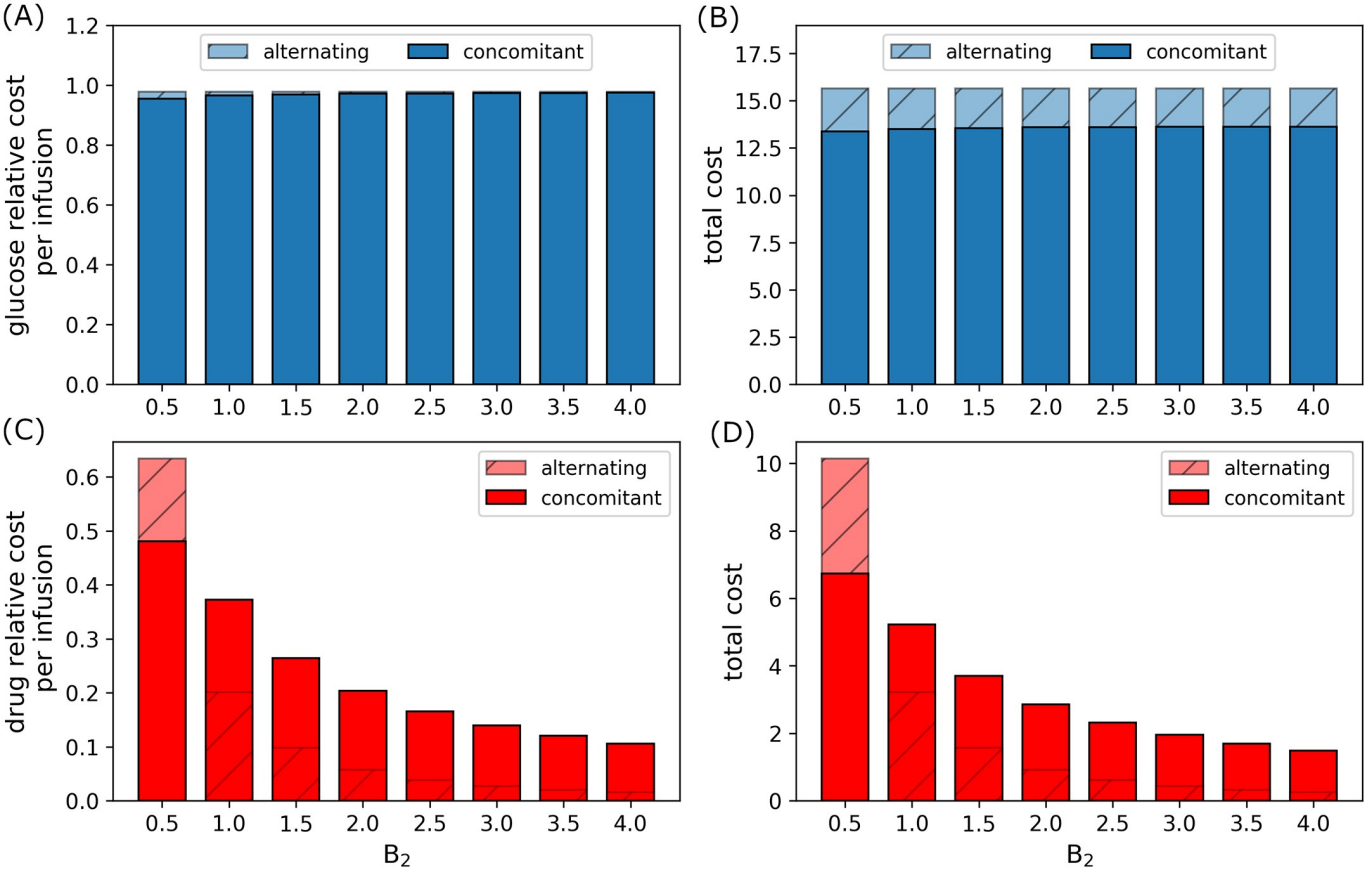
Relative administration cost for varying *B*_2_. Relative glucose administration (A) cost per infusion and (B) total cost, and relative drug administration (C) cost per infusion and (D) total cost incurred for a period of 168*h* (7*d*) for concomitant and alternating controls with increasing *B*_2_.

Figs 15 and 16 reflect the relative cost per infusion and total administration cost of glucose and drug infusions incurred under concomitant and alternating controls. With fixed drug administration cost (*B*_2_ = 1.0), relative glucose cost per infusion and total administration cost increases as *B*_1_ increases. Note that alternating control cost for glucose is always higher than concomitant infusions, see Fig 15(A) and 15(B). The relative and total administration cost for concomitant infusion slightly increase as compared to alternating control as *B*_1_ increases. Again, alternating control incur more cost for higher glucose administration cost as illustrated in Fig 15(C) and 15(D). On the other hand, when *B*_1_ = 1.0 and drug administration cost (*B*_2_) increases, the relative glucose cost per infusion and total administration cost is almost constant. This can be seen in Fig 16(A) and 16(B). Again, relative total cost for alternating control is higher than that of concomitant control. Further, as *B*_2_ increases, relative drug cost per infusion and total administration cost decreases (Fig 16(C) and 16(D)), since total drug dosage also decreases as shown in Fig 14D. In this case, it can be seen that drug administration cost for alternating control is generally lower than that of concomitant control.

**Fig 17:**
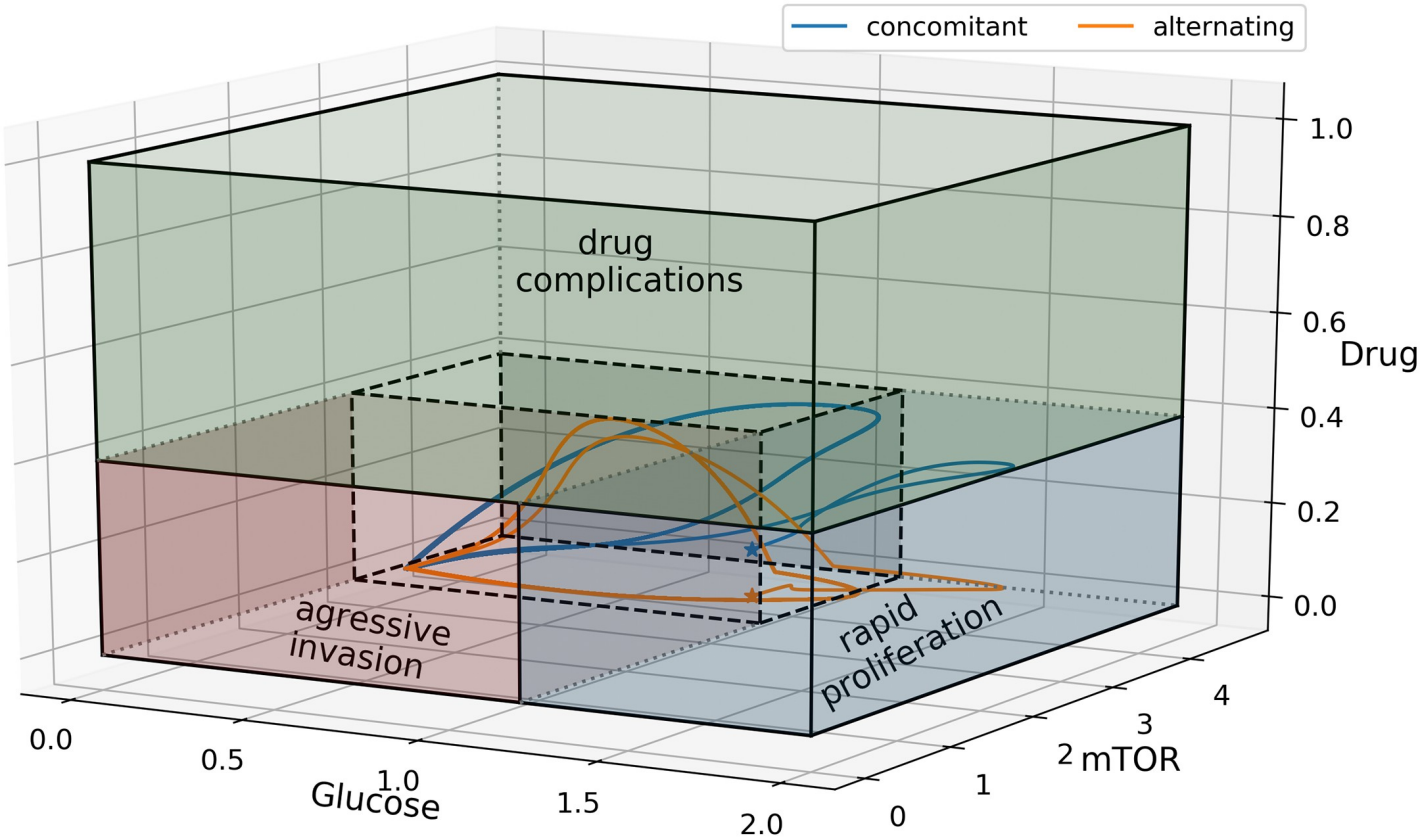
Glucose–mTOR–drug dynamics for concomitant and alternating controls. Concomitant (blue) and alternating (orange) control trajectories are confined in a smaller region avoiding aggressive invasion, rapid proliferation, and unwanted drug complications.

Observe that in Fig 17, both concomitant (blue) and alternating (orange) control trajectories in glucose–mTOR–drug space are restricted in a smaller region avoiding aggressive invasion, rapid proliferation, and unwanted drug complications. Both strategies achieved the goal of keeping mTOR (miR-451) up-regulated inducing cell proliferation and thus avoiding aggressive cell migration. It is important to note that under these proposed optimal control infusions, glucose and drug levels are regulated to prevent excessive cell division and tumor growth. As a consequence, these strategies suggest safer infusion administration preventing hyperglycemia for diabetic patients and risk of drug complications.

Table 3 provides the average frequency, dosage and relative cost of concomitant and alternating control strategies where *B*_1_ = *B*_2_ = 1.0 and simulation time is 168*h* (7*d*).

**Table 3:** Summary for concomitant and alternating controls.

		Concomitant	Alternating
frequency	glucose infusion	0.080297	0.092806
	drug infusion	0.080297	0.092806
glucose dose	per infusion	2.114161	2.135903
	total	29.598253	34.174444
drug dose	per infusion	1.365346	1.044772
	total	19.114839	16.716352
glucose relative cost	per infusion	0.964683	0.978384
	total	13.505558	15.65414
drug relative cost	per infusion	0.372771	0.201441
	total	19.114839	16.716352

## Conclusion

The periodic switching behavior of glioblastoma cells between proliferation and invasion phases is highly influenced by fluctuating glucose levels [14, 17]. In response to high glucose supply, miR-451 and mTOR are up-regulated and AMPK complex is down-regulated inducing cell growth. On the contrary, low glucose level up-regulates AMPK complex, down-regulating miR-451 and mTOR, promoting cell migration [15]. The mutual antagonistic mechanism between miR-451 (mTOR) and AMPK complex and the cell’s strategic metabolic adaptation support the survival of cancer cells even in a nutrient-deprived microenvironment [14, 55].

In addition to rapid proliferation of glioblastoma cells, aggressive invasion to the surrounding tissue is a major cause of treatment failure. Despite advances in medical imaging technology such as MRI and PET, glioblastoma cells can spread beyond detection leading to tumor recurrence within 2 to 3 *cm* of the resection cavity even after surgical removal of a malignant glioblastoma [29]. These glioma cells are capable to deform cell membrane and nucleus for cell infiltration through a narrow gap between normal glial cells in brain tissue by upregulation of myosin II along with actin bundles [26, 56]. While exact migratory patterns are still poorly understood, these invasive glioma cells prefer white matter and blood vessels [26, 57] with a wide range of speeds in the range of 5-80 *μm*/*h* [58–61] showing sometimes saltatory patterns in the migration direction in brain [58]. Prediction of tumour invasion directions in nearby tissue may help define exact boundaries of focal treatments (surgery or radiosurgery), preventing future growth and recurrence [57]. For example, medical doctors could determine specific locations for radiation target volumes, not just using a rough estimate of 2-3 *cm* in all directions as a guidance as commonly done today [57].

Assuming that migratory cells are localized near the surgery site [16], one possible approach is to keep the cells in its proliferative phase preventing them from invading brain tissue in a combination with transport of therapeutic drugs near blood vessels [18]. As a result, the tumor mass will be visible for a succeeding surgery while killing proliferative tumor cells at the blood sites.

In this study, we considered the intracellular dynamics of the miR-451–AMPK-mTOR-cell cycle signaling pathway model developed recently by Kim *et al*. [40]. Incorporated in the model is a drug component which blocks the inhibitory pathway of mTOR by AMPK complex. This drug targets up-regulation of mTOR activities enhancing cell proliferation. The focus of the current work is to regulate up-stream signaling pathway via glucose infusion activating miR-451, and control the down-stream pathway to cell cycle via drug infusion enhancing mTOR activities. Optimal control problem is formulated with the goal of keeping high levels of miR-451 and mTOR to induce cell growth and reduce invasion to the surrounding tissues. In the framework of optimal control theory, two administration strategies are explored to achieve the goal with minimal cost incurred in glucose and drug administrations. The control strategies investigated in this study are (1) concomitant infusion control and, and (2) alternating glucose and drug infusion control. Both strategies are able to switch on the proliferative mode of glioblastoma cells and turn off its migratory mode. Cell cycle is regulated with fewer mass divisions restricting rapid growth. Numerical results show that concomitant control had fewer infusions, lesser glucose dosage and cheaper administration cost. However, when glucose and drug poses unwanted chemical reactions during concurrent administration, alternating control would be beneficial with lower drug amount usage.

The mathematical models of the miR-451-AMPK-mTOR core control [14, 16–18, 40] are based on a ‘go-or-grow’ hypothesis and supporting experiments in GBM cells [8, 15, 19]. However, the range of bistable behavior indicates a ‘go-and-grow’ program which has been proposed for other cancers too [62]. In the glioma cases, ‘go-or-grow’ hypothesis has been long suggested [29, 55, 63–66] in addition to miR-451-induced ‘go-or-grow’ mechanism [8, 15, 19]. Glioma consists of a bulky, proliferative core in the center and highly invasive individual cells in the outer rim [55, 63, 64, 67] and sequential transition between proliferative and invasive phenotypes characterizes tumor progression and may lead to faster growth [14]. At least at the microscopic level, these migration/proliferation phenotypes appear to be mutually exclusive characteristics at different time frames [64], as suggested by in vivo imaging data of glioma cells migrating in a saltatory fashion [58]. Glioma cells was shown to pause for a short period of time (∼an hour) for cell division before the daughter cells begin to move again [58]. Gal *et al*. [68] further experimentally observed that this phenotypic switch can happen under different extracellular environment: (i) proliferative type in soft agar via activation of Myc signaling pathways in response to hepatocyte growth factor (HGF); and (ii) infiltrative type in Matrigel through Ras signaling pathways in response to the same HGF. Recently, Dhruv *et al*. [69] also affirmed the role of activation of key molecules in dichotomy between proliferation and invasion: c-Myc and NF*κ*B in the proliferative core and radially dispersed, invasive region of GBM tumors, respectively. Glial cells can also interact with GBM tumor cells for phenotypic switch of GBM cells between cell migration and active proliferation [66]. Although these experiment and mathematical modeling support the ‘go-or-grow’ hypothesis, it is not certain if alternative mechanism such as ‘go-and-grow’ occurs in such a heterogenous TME in real patients [64]. Mansury *et al*. [70] for instance illustrated that individual glioma cell in a mixed group of proliferative and invasive phenotypes can depend on genotype of counter part and tumor microenvironment. In a similar conceptual studies on epithelial-to-mesenchymal transition (EMT) and mesenchymal-to-epithelial transition (MET) [45] can give a hint on the complexities of this complex regulation of those two phenotypes and glioma in TME might not posses the simple ‘go-or-grow’ dogma. Increasing number of evidences now suggest that the ‘go-or-grow’ model has similar molecular basis with EMT [64]. For instance, HGF and TGF*β* are major regulators of EMT, and these also provide strong stimuli of GBM invasion [71, 72] and, upregulation of MET and CD44 activities, as well as an activated NF*κ*B signaling pathway, was reported in both mesenchymal GBM and metastatic cancer [73–76]. All these experimental observations suggest that metastatic epithelial cancers and mesenchymal GBM drive common mechanisms that regulate the phenotypic transition between invasion and proliferation, suggesting the possibility of the ‘go-and-grow’ mechanism. The EMT paradigm in GBM has not been extensively studied as relevant to the progression of the disease due to different origin and rare metastasis of glioma [77, 78]. Further studies and experimentation need to be done for better understanding of the fundamental principles.

In this paper, we did not take into account key microenvironmental factors such as endogenous immune dynamics including NK cells [79] and M1/M2 macrophages [72], other major signaling networks [80, 81] such as E2F and Myc [11, 82], angiogenesis [80, 83], biophysical interaction between tumor cells and blood vessels [80], ECM remodeling for therapy [81, 84–86], or growth factors [87, 88] such as epidermal growth factors [72, 89, 90], fibroblast growth factors [91], transforming growth factor-*β* [72, 92], and CSF-1 [72, 93, 94], that may play critical roles in proliferation, progress, aggressive invasion of gliomas and development of anti-cancer strategies [95]. For example, endogenous NK cells may interfere oncolytic virus combination therapy in GBM while exogenous NK cells increase anti-tumor efficacy [79], illustrating the complex nature of tumor microenvironment. Recently, mTOR was considered to be a master regulator of cell growth and recognized as a good therapeutic target for therapies in glioblastoma [96]. A recent study found that withaferin A and temozolomide can induce apoptosis and reduce drug resistance by G2/M cell cycle arrest through intrinsic and extrinsic apoptotic signaling pathways [97]. More detailed analysis and experiments on Akt/mTOR/PI3K are necessary. Interestingly, radiation was shown to indirectly promote the export of lactate into the extracellular space and inhibition of AMPK/NF*κ*B signaling pathways were involved in radiation-induced invasion of cancer cells [98]. On the other hand, M2 microglia/macrophages induce matrix remodeling and glioma cell invasion [72, 99–101]. Since PI3K signaling was shown to contribute to M2-polarization of these tumor associated macrophages (TAMs) [102] and PI3K binding to CSF1R was shown to enhance spreading of macrophages, thus promoting glioma cell invasion [103]. Therefore, better understanding of these PI3K-mTOR-CSF1R signaling networks in macrophages would lead to development of blocking aggressive infiltration of cancer cells. Signaling pathways of apoptosis and necroptosis are important parts of oncolytic virus (OVs) therapy [104, 105]. Tumor extracellular matrix (ECM) plays a significant role in regulation of glioma invasion in brain tissue as well as OV spread [106]. For example, CSPGs, one of major tumor ECM in brain can characterize the invasive and non-invasive gliomas in a complex TME where microglia and astrocytes mechanically interact with CSPGs and tumor cells [40, 107–109]. Hybrid models [56, 110–112] and its associated optimal control strategies can be adapted to take into account this important intracellular signaling as well as cell population dynamics and tissue dynamics. Better understanding of various roles of these components in tumor microenvironment may provide better anti-invasion strategies of glioma cells.

However, our mathematical model in this work is a first step toward further experimental/clinical investigation and more optimal anti-invasion strategies of GBM by incorporating these microenvironmental components. We will address these issues in future work.

## Supporting information

S1 Appendix Optimal control problem. Formulation of the optimal control problem for concomitant and alternating glucose and drug infusion.
(PDF)
