# Supplementary material for: Strategies in regulating glioblastoma signaling pathways and anti-invasion therapy
Source: PLoS One. 2019 Apr 22;14(4):e0215547. doi: 10.1371/journal.pone.0215547 (PMC6476530; doi:10.1371/journal.pone.0215547)
Supplement: S1 Appendix — Formulation of the optimal control problem for concomitant and alternating glucose and drug infusion. (PDF) [file pone.0215547.s001.pdf]

S1 Appendix for "Strategies in regulating glioblastoma signaling pathways and anti-invasion therapy"

Eunok Jung[1], Aurelio A. de los Reyes V[1,2], Kurt Jan A. Pumares[2], Yangjin Kim [1,3*]

1 Department of Mathematics, Konkuk University, Seoul, Republic of Korea
2 Institute of Mathematics, University of the Philippines Diliman, Quezon City, Philippines
3 Mathematical Biosciences Institute and Department of Mathematics, Ohio State University, Columbus, Ohio, United States of America
*e-mail: ahyouhappy@konkuk.ac.kr

Optimal control problem

Concomitant glucose and drug infusion control

In this strategy, glucose and drug are simultaneously administered on the same time interval, $[t_0, t_1]$. Since glucose targets the up-stream signaling pathway by activating miR-451 levels and the drug aims to regulate the down-stream mTOR activities leading to cell cycle, the goal is to maximize the objective functional

$$J_{\text{con}}\big(u_G(t), u_D(t)\big) = \int_{t_0}^{t_1} \left(M(t) + R(t) - \left(\frac{B_1}{2} u_G(t)^2 + \frac{B_2}{2} u_D(t)^2 \right) \right) dt, \tag{1}$$

over

$$\Omega_{\text{con}} = \left\{ u_G(t), u_D(t) \in \mathscr{L}(t_0, t_1) \mid 0 \le u_G(t), u_D(t) \le u_{\max}, t \in [t_0, t_1] \right\}, \tag{2}$$

subject to

$$\begin{aligned}
\frac{dG}{dt} &= u_G(t) - \mu_G, \\
\frac{dM}{dt} &= G + \frac{\ell_1 \ell_2^2}{\ell_2^2 + \alpha A^2} - M, \\
\frac{dA}{dt} &= \frac{1}{\varepsilon_1} \left(S_1 + \frac{\ell_3 \ell_4^2}{\ell_4^2 + \beta M^2} - A \right), \\
\frac{dR}{dt} &= \frac{1}{\varepsilon_2} \left(S_2 + \frac{\ell_5 \ell_6^2}{\ell_6^2 + \gamma e^{-D} A^2} - R \right), \\
\frac{dD}{dt} &= u_D(t) - \mu_D, \qquad\qquad (3) \\
\frac{d[CycB]}{dt} &= k_1 - \left(k_2' + k_2''[Cdh1] + [p27/p21][HIF] \right) [CycB], \\
\frac{d[Cdh1]}{dt} &= \frac{(k_3' + k_3''[p55cdc_A])\,(1 - [Cdh1])}{J_3 + 1 - [Cdh1]} - \frac{k_4 [mass_s][CycB][Cdh1]}{J_4 + [Cdh1]},
\end{aligned}$$

$$\frac{d[p55cdc_T]}{dt} = k_5' + k_5'' \frac{([CycB][mass_s])^n}{J_5^n + ([CycB][mass_s])^n} - k_6[p55cdc_T],$$

$$\frac{d[p55cdc_A]}{dt} = \frac{k_7[Plk1]\left([p55cdc_T] - [p55cdc_A]\right)}{J_7 + [p55cdc_T] - [p55cdc_A]} - \frac{k_8[Mad][p55cdc_A]}{J_8 + [p55cdc_A]} - k_6[p55cdc_A],$$

$$\frac{d[Plk1]}{dt} = k_9[mass_s][CycB]\left(1 - [Plk1]\right) - k_{10}[Plk1],$$

$$\frac{d[mass]}{dt} = \mu[mass]\left(1 - \frac{[mass]}{m^*}\right),$$

where

$$[mass_s] = [mass] + \frac{\zeta_1\left(\frac{1}{R}\right)^{n_1}}{K_m^{n_1} + \left(\frac{1}{R}\right)^{n_1}}, \qquad [HIF] = \frac{\zeta_2\left(\frac{1}{K}\right)^{n_2}}{K_H^{n_2} + \left(\frac{1}{K}\right)^{n_2}}, \qquad \mu = \mu^+ + \epsilon\hat{\mu}. \tag{4}$$

It should be noted that maximizing the objective functional entails finding control glucose $u_G^*(t)$ and drug $u_D^*(t)$ infusions that up-regulates miR-451 (M) and mTOR (R) above its respective threshold values. At the same time, intravenous administration costs should be minimized. We have the following theorem:

Theorem 1. *There exist optimal controls $u_G^*(t)$ and $u_D^*(t)$, and corresponding solutions $G^*(t)$, $M^*(t)$, $A^*(t)$, $R^*(t)$, $D^*(t)$, $[CycB]^*$, $[Cdh1]^*$, $[p55cdc_T]^*$, $[p55cdc_A]^*$, $[Plk1]^*$, and $[mass]^*$ that maximize the objective functional* (1) *over* (2)*. Given this optimal solution, there exist adjoint equations satisfying*

$$\begin{aligned}
\lambda_1' &= \lambda_1\mu_G - \lambda_2 \\
\lambda_2' &= -1 + \lambda_2 + \lambda_3 \frac{1}{\varepsilon_1} \frac{2\ell_3\ell_4^2\beta M}{\left(\ell_4^2 + \beta M^2\right)^2} \\
\lambda_3' &= \lambda_2 \frac{2\ell_1\ell_2^2\alpha A}{\left(\ell_2^2 + \alpha A^2\right)^2} + \lambda_3\frac{1}{\varepsilon_1} + \lambda_4\frac{1}{\varepsilon_2}\frac{2\ell_5\ell_6^2\gamma e^{-D}A}{\left(\ell_6^2 + \gamma e^{-D}A^2\right)^2} \\
\lambda_4' &= -1 + \lambda_4\frac{1}{\varepsilon_2} + \frac{\partial[mass_s]}{\partial R}\Bigg(\lambda_7\frac{k_4[CycB][Cdh1]}{J_4 + [Cdh1]} - \lambda_8\frac{k_5'' n J_5^n\left([CycB][mass_s]\right)^n}{[mass_s]\left(J_5^n + ([CycB][mass_s])^n\right)^2} \\
&\qquad - \lambda_{10}k_9[CycB]\left(1 - [Plk1]\right)\Bigg) \\
\lambda_5' &= -\lambda_4\frac{1}{\varepsilon_2}\frac{\ell_5\ell_6^2\gamma e^{-D}A^2}{\left(\ell_6^2 + \gamma e^{-D}A^2\right)^2} + \lambda_5\mu_D \\
\lambda_6' &= \lambda_6\left(k_2' + k_2''[Cdh1] + [p27/p21][HIF]\right) + \lambda_7\frac{k_4[mass_s][Cdh1]}{J_4 + [Cdh1]} \\
&\qquad - \lambda_8\frac{k_5'' J_5^n n\left([CycB][mass_s]\right)^n}{[CycB]\left(J_5^n + ([CycB][mass_s])^n\right)^2} - \lambda_{10}k_9[mass_s]\left(1 - [Plk1]\right) \\
\lambda_7' &= \lambda_6 k_2''[CycB] + \lambda_7\left(\frac{J_3\left(k_3' + k_3''[p55cdc_A]\right)}{\left(J_3 + 1 - [Cdh1]\right)^2} + \frac{k_4J_4[CycB][mass_s]}{\left(J_4 + [Cdh1]\right)^2}\right) \\
\lambda_8' &= \lambda_8k_6 - \lambda_9\frac{k_7J_7[Plk1]}{\left(J_7 + [p55cdc_T] - [p55cdc_A]\right)^2}
\end{aligned} \tag{5}$$

$$
\begin{aligned}
\lambda_9' &= -\lambda_7 \frac{k_3''\left(1-[Cdh1]\right)}{J_3+1-[Cdh1]} + \lambda_9\Bigg(k_6 + \frac{k_7 J_7 [Plk1]}{\left(J_7+[p55cdc_T]-[p55cdc_A]\right)^2} \\
&\qquad + \frac{k_8 J_8 [Mad]}{\left(J_8+[p55cdc_A]\right)^2}\Bigg) \\
\lambda_{10}' &= -\lambda_9 \frac{k_7\left([p55cdc_T]-[p55cdc_A]\right)}{J_7+[p55cdc_T]-[p55cdc_A]} + \lambda_{10}\left(k_9[CycB][mass_s]+k_{10}\right) \\
\lambda_{11}' &= -\frac{\partial H}{\partial [mass]} \;=\; \lambda_7 \frac{k_4[CycB][Cdh1]}{J_4+[Cdh1]} - \lambda_8 \frac{k_5'' n J_5^n \left([CycB][mass_s]\right)^n}{[mass_s]\left(J_5^n+\left([CycB][mass_s]\right)^n\right)^2} \\
&\qquad - \lambda_{10} k_9 [CycB]\left(1-[Plk1]\right) + \lambda_{11}\mu\Bigg(\frac{2[mass]}{m^*}-1\Bigg)
\end{aligned}
$$

where

$$
\frac{\partial [mass_s]}{\partial R} = \frac{-\zeta_1 n_1 K_m^{n_1}\left(\dfrac{1}{R}\right)^{n_1}}{R\left(K_m^{n_1}+\left(\dfrac{1}{R}\right)^{n_1}\right)^2}, \tag{6}
$$

with transversality conditions

$$
\lambda_i(t_1) = 0 \quad \textit{for} \quad i = 1, 2, \ldots, 11. \tag{7}
$$

Furthermore,

$$
\begin{aligned}
u_G^*(t) &= \min\left(u_{\max}, \max\left(u_{\min}, \frac{\lambda_1}{B_1}\right)\right), \\
u_D^*(t) &= \min\left(u_{\max}, \max\left(u_{\min}, \frac{\lambda_5}{B_2}\right)\right).
\end{aligned} \tag{8}
$$

Proof. The convexity of the integrand of objective functional guarantees the existence of optimal controls $u_G^*(t)$ and $u_D^*(t)$. Applying Pontryagin's Maximum principle converts our maximization problem into maximizing the Hamiltonian given by

$$
H = M + R - \frac{B_1}{2}u_G^2 - \frac{B_2}{2}u_D^2 + \sum \lambda_i f_i, \tag{9}
$$

where λ_i's are so-called adjoints and f_i's are the right hand side of Eq (3) for $i = 1, 2, \ldots, 11$. The following adjoint equations and transversality conditions are obtained:

$$
\begin{aligned}
\lambda_1' &= -\frac{\partial H}{\partial G}, & \lambda_1(t_1) &= 0, \\
\lambda_2' &= -\frac{\partial H}{\partial M}, & \lambda_2(t_1) &= 0, \\
\lambda_3' &= -\frac{\partial H}{\partial A}, & \lambda_3(t_1) &= 0, \\
\lambda_4' &= -\frac{\partial H}{\partial R}, & \lambda_4(t_1) &= 0, \\
\lambda_5' &= -\frac{\partial H}{\partial D}, & \lambda_5(t_1) &= 0, \\
\lambda_6' &= -\frac{\partial H}{\partial [CycB]}, & \lambda_6(t_1) &= 0,
\end{aligned} \tag{10}
$$

$$\lambda_7' = -\frac{\partial H}{\partial [Cdh1]}, \qquad \lambda_7(t_1) = 0,$$
$$\lambda_8' = -\frac{\partial H}{\partial [p55cdc_T]}, \qquad \lambda_8(t_1) = 0,$$
$$\lambda_9' = -\frac{\partial H}{\partial [p55cdc_A]}, \qquad \lambda_9(t_1) = 0,$$
$$\lambda_{10}' = -\frac{\partial H}{\partial [Plk1]}, \qquad \lambda_{10}(t_1) = 0,$$
$$\lambda_{11}' = -\frac{\partial H}{\partial [mass]}, \qquad \lambda_{11}(t_1) = 0.$$

After differentiating H with respect to the control u_G and u_D and considering the bounds yield the characterization of the controls given by Eq (8). □

Alternating glucose and drug infusion control

This strategy proposes alternating glucose and drug intravenous infusions. Recall that glucose directly regulates miR-451 and mTOR activities in the up-stream and down-stream signalling pathway, respectively. The idea is to have glucose infusion only on $[t_0, t_1]$ and drug infusion on $[t_1, t_2]$. Hence, the goal is to maximize the objective functional

$$J_{\text{alt}}\big(u_1(t), u_2(t)\big) = \int_{t_0}^{t_1} \left(M(t) + R(t) - \frac{B_1}{2} u_G(t)^2 \right) dt + \int_{t_1}^{t_2} \left(R(t) - \frac{B_1}{2} u_D(t)^2 \right) dt, \tag{11}$$

over

$$\begin{aligned} \Omega_{\text{alt}} = \{ & u_G(t) \in \mathscr{L}(t_0, t_1), u_D(t) \in \mathscr{L}(t_1, t_2) \mid \\ & 0 \le u_G(t) \le u_{\max}, t \in [t_0, t_1], \quad 0 \le u_D(t) \le u_{\max}, t \in [t_1, t_2] \}, \end{aligned} \tag{12}$$

subject to system (3) where $u_D = 0$ for $t \in [t_0, t_1]$ (i.e., no drug infusion) , and $u_G = 0$ for $t \in [t_1, t_2]$ (i.e., no glucose infusion), with auxilliary equations (4). It is assumed that the drug in consideration only influences the down-ward signaling pathway to the cell cycle. We have the following theorem:

Theorem 2. *There exist optimal controls $u_G^*(t)$ for $t \in [t_0, t_1]$ and $u_D^*(t)$ for $t \in [t_1, t_2]$, and corresponding solutions $G^*(t)$, $M^*(t)$, $A^*(t)$, $R^*(t)$, $D^*(t)$, $[CycB]^*$, $[Cdh1]^*$, $[p55cdc_T]^*$, $[p55cdc_A]^*$, $[Plk1]^*$, and $[mass]^*$ that maximize the objective functional* (11) *over* (12)*. Given this optimal solution, there exist adjoint equations satisfying Eq* (5) *with transversality conditions*

$$\lambda_i(t_j) = 0 \quad \textit{for} \quad i = 1, 2, \ldots, 11, \quad j = 1, 2. \tag{13}$$

Furthermore,

$$\begin{aligned} u_G^*(t) &= \min\left(u_{\max}, \max\left(u_{\min}, \frac{\lambda_1}{B_1}\right)\right) \quad \textit{for} \quad t \in [t_0, t_1], \\ u_D^*(t) &= \min\left(u_{\max}, \max\left(u_{\min}, \frac{\lambda_5}{B_2}\right)\right) \quad \textit{for} \quad t \in [t_1, t_2]. \end{aligned} \tag{14}$$

The proof of the above theorem follow analogously from the previous theorem with modifications on Hamiltonian, adjoint and optimality equations accounting for different objective functional for $t \in [t_0, t_1]$ and $t \in [t_1, t_2]$.

In the following simulation results, the weight parameters $B_1 = B_2 = 1.0$, the simulation time is 168 hours (7 days), and maximum dosage rate $u_{\max} = 1.0$. Fig A illustrates the optimal glucose dosage rate $u_G^*(t)$ and corresponding time evolution of glucose concentrations for concomitant (blue curves) and alternating controls (orange curves). It can be seen that alternating control yields more glucose infusion over the simulation time and the glucose profile is shifted to the left. In Fig B, optimal drug dose rates $u_D^*(t)$ and corresponding time evolution of drug concentrations for concomitant (blue curves) and alternating controls (orange curves) are depicted. Notice the difference in infusion times and drug concentration dynamics. It is shown in Fig C that both strategies are able to restrict miR-451 and mTOR above their respective threshold values, and AMPK complex levels below its threshold. Fig D displays the trajectory of concomitant and alternating controls in 3D spaces showing different perspectives.

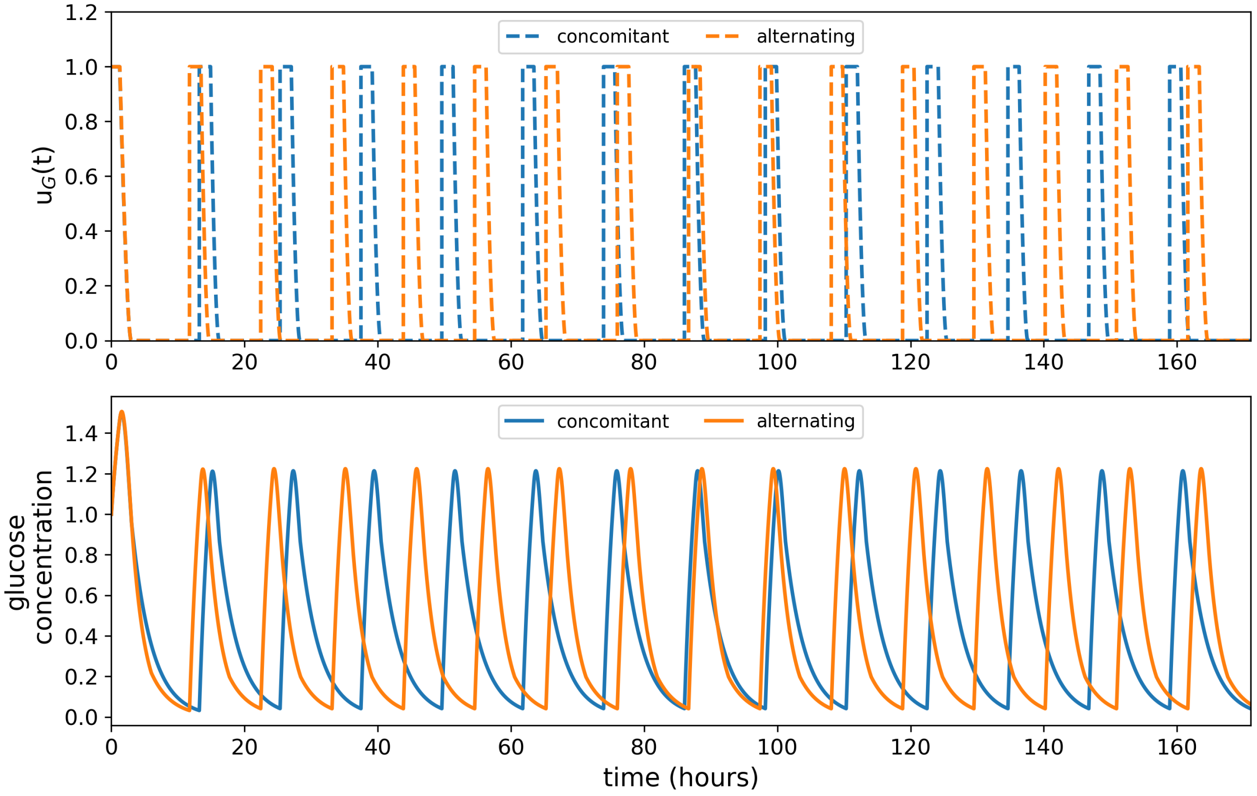

Figure A: **Glucose concentration profiles.** Glucose infusion protocol and corresponding concentrations under concomitant and alternating controls over 168h (7d) period.

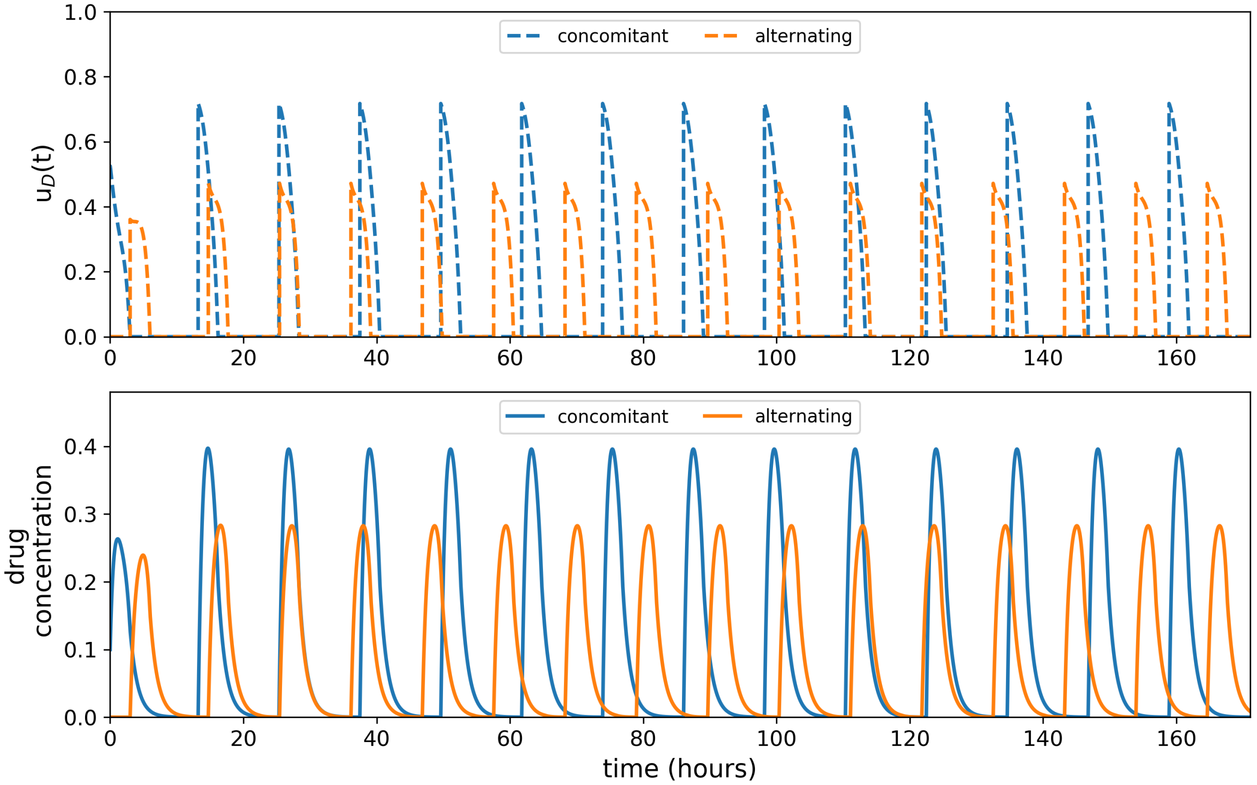

Figure B: Drug concentration profiles. Drug infusion protocol and corresponding concentrations under concomitant and alternating controls over 168h (7d) period.

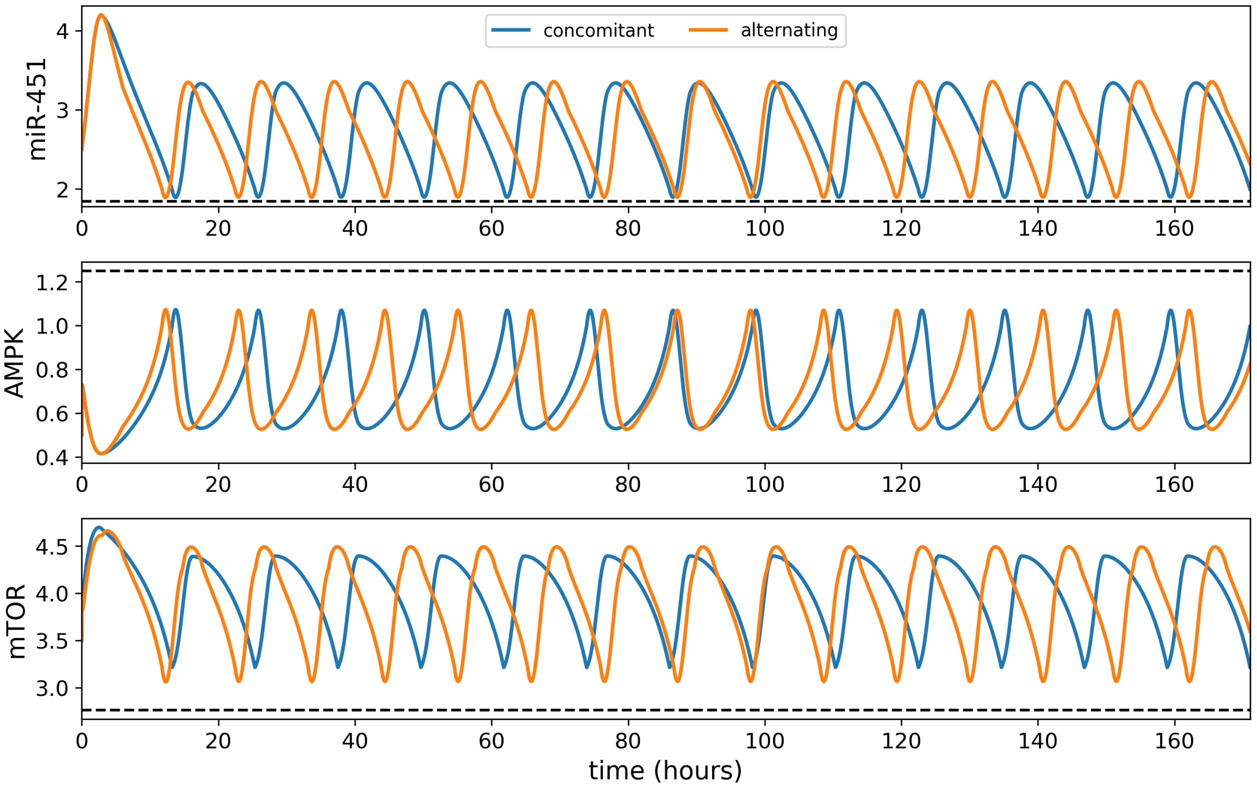

Figure C: **miR-451, AMPK, mTOR levels.** Concentration profiles of miR-451, AMPK, and mTOR under concomitant and alternating controls over 168h (7d) period.

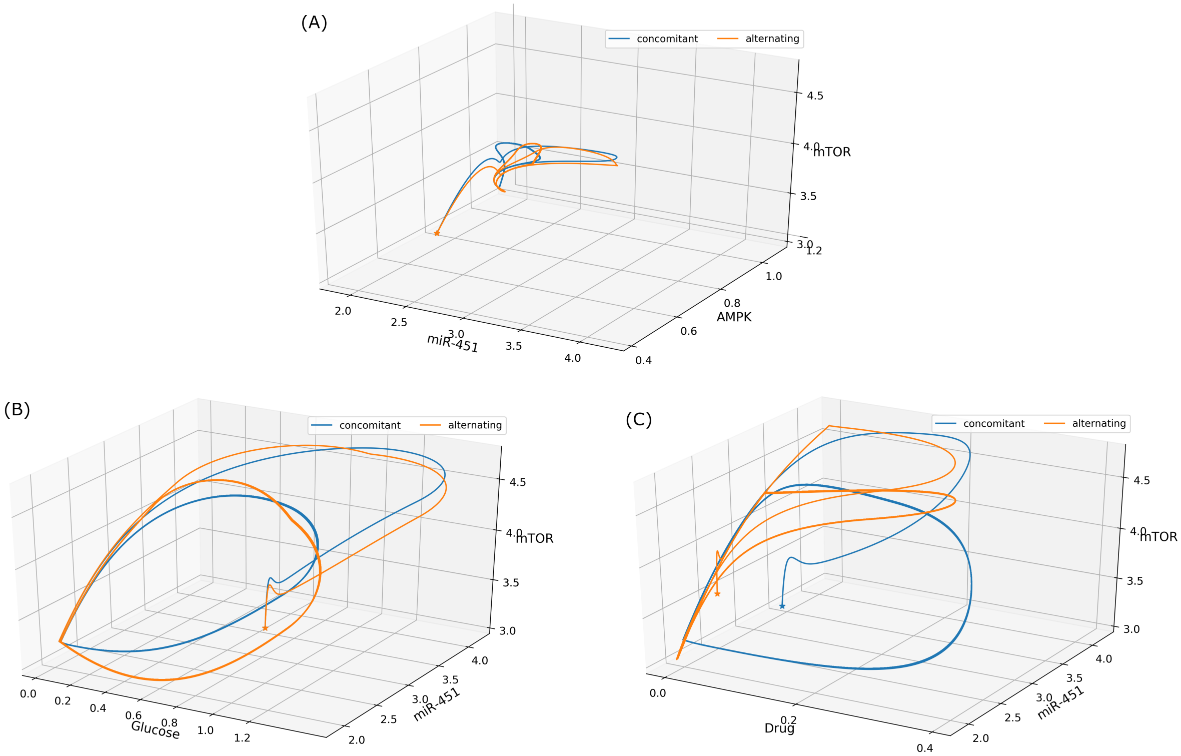

Figure D: **3D trajectories.** Concomitant and alternating control trajectories in (A) miR-451–AMPK-mTOR space, (B) glucose–miR-451–mTOR space, and (C) drug–miR-451–mTOR space.
